# Incidental findings from panoramic radiographs: A systematic review with meta-analysis

**DOI:** 10.1007/s00784-026-07148-4

**Published:** 2026-09-24

**Authors:** Anastasia Imboden-Ilia, Heba Awaad, Emmanouil Inglezos, Theodore Eliades, Spyridon N. Papageorgiou

**Affiliations:** 1 Clinic of Orthodontics and Pediatric Dentistry, Center for Dental Medicine, Faculty of Medicine, University of Zurich, Plattenstrasse 11, 8032 Zurich, Switzerland; 2 Private Clinic, Basel, Switzerland; 3 Private Practice, Zurich, Switzerland

**Keywords:** Dentistry, Radiography, Panoramic radiograph, Incidental finding, Pathology, Prevalence

## Abstract

**Purpose:**

The use of radiographs is widely spread, both among general dentists and specialists, since it provides a view of the oral and perioral tissues using a relatively easy-to-acquire and low-dose image. The aim of this systematic review was to critically appraise and summarize evidence from clinical studies on the prevalence of incidental findings from panoramic radiographs.

**Method:**

Systematic literature searches without restrictions were undertaken in eight databases from inception up to January 2026 for studies reporting on incidental findings from panoramic radiographs of any dental patients. After duplicate study selection, data extraction, and risk-of-bias assessment using a custom tool based on the Joanna Briggs Institute’s tool for prevalence studies, random-effects meta-analyses were performed, followed by subgroup, meta-regression, and sensitivity analyses.

**Results:**

Twenty-five studies covering a total of 66 882 patients (44.1% male; average age 28.9 years) were included. The prevalence of incidental findings ranged from 5.2% to 88.1%, with average distribution prevalences between 22.9% and 53.1%, but heterogeneity was high across studies. Incidental findings were most often related to airways (30.9%), followed by teeth (12.2%), soft-tissue calcifications (9.4%), spine (7.4%), radiopacities (7.1%), and temporomandibular joint (1.8%). However, several methodological issues were present in the included studies (incomplete reporting of patient- or radiography-related details, incomplete categorization and reporting of the severity of findings, small sample sizes, and issues with research transparency).

**Conclusion:**

Evidence indicates that incidental findings are common in panoramic radiographs of dental patients. Although many incidental findings are clinically insignificant, some may have important implications. When a dental panoramic image is justified, it should be adequately assessed for incidental findings by either a specialist oral and maxillofacial radiologist or a dentist with appropriate training and experience.

**Supplementary Information:**

The online version contains supplementary material available at https://doi.org/10.1007/s00784-026-07148-4.

## Introduction

Dental panoramic radiography remains one of the most widely used imaging modalities in dental practice because it provides a comprehensive overview of the dentition, jaws, temporomandibular joints, maxillary sinuses, and adjacent craniofacial structures within a single examination [1–3]. Owing to its broad field of view, relatively low radiation dose, and cost-effectiveness, panoramic imaging is routinely employed for orthodontic assessment, third molar evaluation, implant planning, oral surgery, and general dental diagnostics [1, 2, 4]. Unlike intraoral radiographs, panoramic radiographs capture numerous anatomical structures beyond the immediate region of clinical interest, creating opportunities to detect abnormalities that may not be apparent during clinical examination alone [3–5]. Consequently, professional and radiological guidelines emphasize that panoramic radiographs should be interpreted systematically and comprehensively, irrespective of the original indication for imaging [1, 2, 6].

Incidental findings are commonly defined as unexpected radiographic observations unrelated to the primary purpose of image acquisition, but that may nevertheless have diagnostic or therapeutic relevance [2, 7]. In dental panoramic radiography, such findings may include developmental dental anomalies, odontogenic and periapical pathology, soft-tissue calcifications, maxillary sinus abnormalities, temporomandibular joint alterations, and other craniofacial findings [3, 4, 8–10]. Although many incidental findings are clinically insignificant, others may influence orthodontic, restorative, surgical, or implant treatment planning. At the same time, some, such as carotid artery calcifications, may indicate previously undiagnosed systemic disease requiring medical evaluation [10–14]. Previous cross-sectional studies have consistently reported incidental findings on panoramic radiographs; however, prevalence estimates vary considerably due to differences in patient populations, referral indications, diagnostic criteria, and assessment methods [3–5, 10, 15].

Despite growing recognition of the clinical importance of incidental findings, the available evidence remains fragmented and difficult to interpret. Individual studies have typically focused on specific populations or selected categories of abnormalities, and substantial variability exists regarding the definition, classification, and reporting of incidental findings [1–3, 5, 7]. To date, no comprehensive systematic review and meta-analysis has synthesized the prevalence and the full spectrum of incidental findings detected on dental panoramic radiographs across different clinical settings and patient populations. Therefore, the aim of the present systematic review and meta-analysis was to evaluate the prevalence of incidental findings on dental panoramic radiographs and to characterize their distribution across anatomical and diagnostic categories.

## Materials and methods

### Protocol and registration

The protocol for this review was developed and registered with PROSPERO before the review’s initiation, and any post hoc deviations were transparently reported (Appendix 1). This review was conducted and reported according to the appropriate chapter of the Joanna Briggs Institute (JBI) Manual for Evidence Synthesis [16].

### Eligibility criteria

The research question for the present review was based on the Participants – Exposure – Comparison – Outcome – Study design (PECOS) framework: What incidental findings can be found in panoramic radiographs of dental patients from observational studies (Appendix 2). Studies of healthy patients of any age, sex, or ethnicity with any anamnestic or clinical justification deemed in need of a panoramic radiograph for diagnostic imaging were eligible for inclusion. If studies reported on mixed samples of children and adults, we used this information, whenever provided, in the analyses.

The condition of interest was any incidental finding from panoramic radiographic images. Any clinical setting was included to increase the applicability/generalizability of the results. Other important outcomes were the prevalence of any specific clinical outcomes, either alone or categorized by the authors of the original studies. We included studies of panoramic radiographs of the head/neck region for diagnostic purposes and excluded other imaging modalities, radiography used for cancer radiotherapy, and radiographs of other body regions. We included studies that assessed panoramic radiographs, conducted either longitudinally or cross-sectionally (in both prospective and retrospective designs). Animal studies, case series/reports, and non-clinical studies (i.e., cadaver studies) were excluded.

### Search strategy

We searched the following electronic general, open access, regional, and grey literature bibliographic databases: MEDLINE (searched via PubMed), Embase, The Cochrane Library (CDSR, CENTRAL, and DARE), Virtual Health Library (including Bibliography Brazilian Dentistry and LILACS), Scopus, and Web of Science (Appendix 3) up to January 2026. Additionally, the Directory of Open Access Journals (DOAJ), Digital Dissertations (searched via UMI ProQuest), the metaRegister of Controlled Trials, the WHO trials search portal, and Google Scholar were searched manually. No restrictions were applied regarding publication year, language, or publication status, and no search filters were applied other than for studies on humans, where available. Hand searching was also performed using the reference and citation lists (via Google Scholar^®^) of the full-text articles that were eligible for inclusion and of relevant systematic reviews.

### Study selection and data extraction

Two review authors (AII and HA) screened the titles and/or abstracts of studies retrieved from the searches and from additional sources (hand searching, reference/citation lists) to identify articles that potentially met the inclusion criteria. The full texts of these potentially eligible studies, as well as those abstracts that did not provide sufficient information to allow decision-making regarding inclusion or exclusion, were retrieved and assessed by one review author (AII), while a second checked the decisions (HA). Any differences between the two reviewers were discussed and settled by consensus and, if needed, by consulting two other authors (EI or TE).

Data collection from the identified reports was conducted using predefined forms that were finalized and piloted by two authors (AII and HA) prior to the completion of the literature searches. Data extraction was undertaken independently and in duplicate, including study design, setting and location, country of origin, number of patients, sex, age, reason for panoramic radiograph acquisition, panoramic radiograph technical details, assessor details, intra- / inter-assessor reliability measures, and reported findings. Discrepancies were resolved as above.

### Risk of bias assessment

The risk of bias within included studies was assessed using JBI’s Critical Appraisal Instrument for Studies Reporting Prevalence Data [17], with appropriate modifications and additions to tailor the tool to the systematic review (Appendix 4) and to the review’s focus, after checking similar reviews.

### Data synthesis

Single-group meta-analysis of proportions and their 95% confidence intervals (CIs) was used to pool data from at least two similar studies reporting on the same incidental finding. As several characteristics related to patient demographics, patient anatomy, assessor experience, or radiograph quality [5, 18] might influence the prevalence of incidental findings, it was judged that a common-effect model would be inappropriate and that a random-effects model to estimate the average distribution of prevalences might be better suited [19]. A one-step logistic-normal generalized linear mixed model with a logit transformation was chosen [20], using Wilson score confidence intervals [21], maximum likelihood estimation for between-study variance [22], and Hartung-Knapp adjustment [23], while proportions were displayed as percentages. Heterogeneity between studies was assessed through visual inspection of a contour-enhanced forest plot (Appendix 1) [24] and through estimation of tau^2^ (absolute heterogeneity) and I^2^ (relative inconsistency). 95% predictions were calculated to incorporate identified heterogeneity and assist in interpreting the meta-analytical estimates by providing a range of expected effects across various future clinical settings [25]. Random-effects meta-regressions and subgroup analyses were performed according to mean age, included age group (underage, adult, mixed), and % of male patients in the study. Reporting bias (including the possibility of small-study effects and publication bias) was assessed through visual inspection of contour-enhanced funnel plots and formally with the Thompson-Sharp test. Sample size (using arbitrary cut-offs [Appendix 1]) was used to run sensitivity analysis as a proxy for precision. All analyses were conducted in R 4.4.1. (R Foundation for Statistical Computing, Vienna, Austria) by one author (SNP), with an open dataset [26], alpha set at 0.05 (except for 0.10 for subgroup and meta-regression analyses) and a two-sided P-value.

### Certainty of evidence

The certainty of the available evidence was not formally addressed, since no guidance exists for the systematic review of prevalence studies. Interpretation of the studies’ results was performed in accordance with their risk of bias.

## Results

### Study selection

**Fig. 1:**
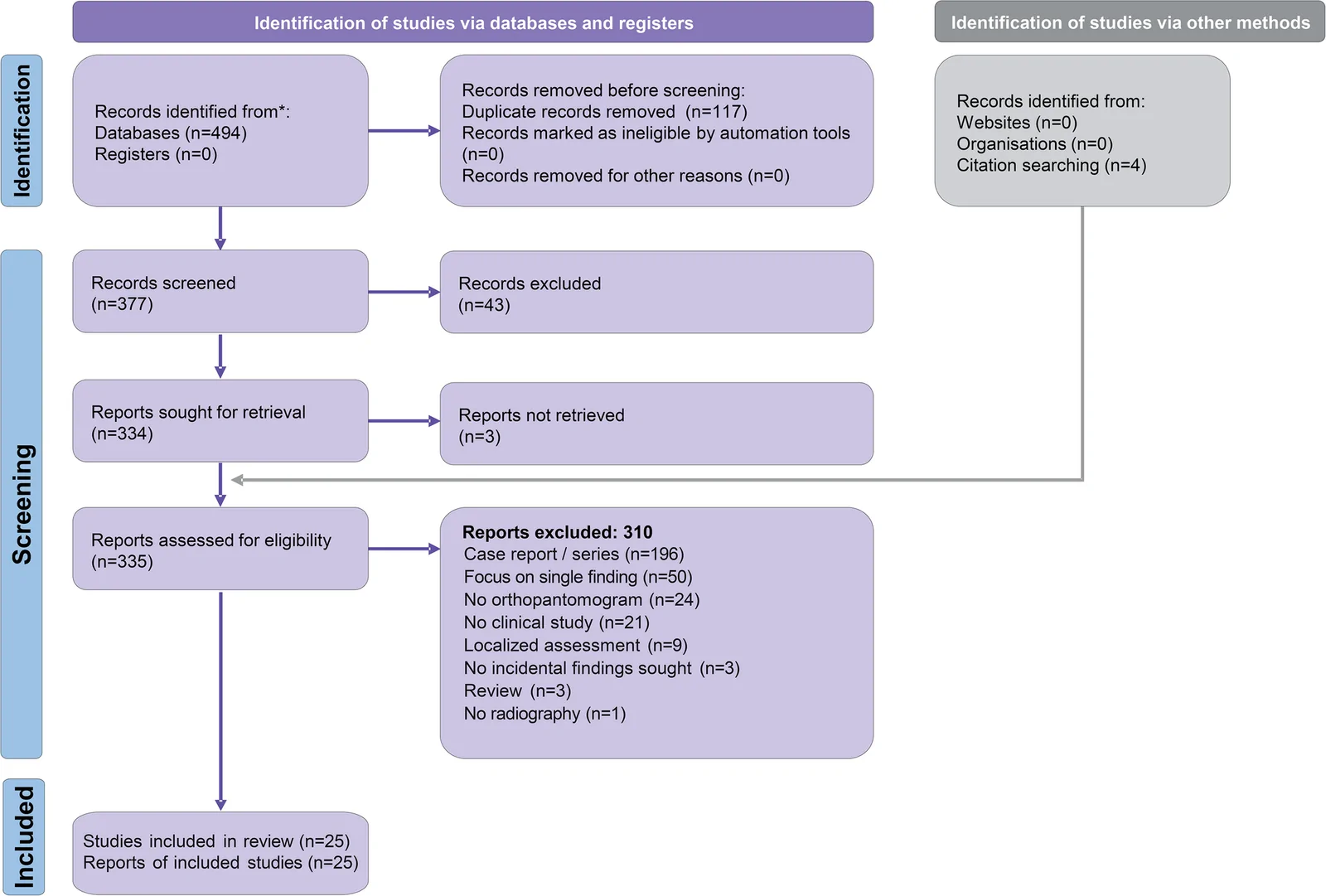
Characteristics of included studies

The electronic records search yielded a total of 494 records, with a search of additional resources (hand-searching) yielding four records. Removal of duplicates resulted in 377 unique records (Appendix 5). After removing obviously non-relevant records from the title/abstract, 334 reports were ultimately checked against the eligibility criteria, and 25 papers reporting on 25 unique studies [3–5, 27–48] were included (Fig. 1).

### Study characteristics

Twenty-four studies were published as journal articles and one as a thesis [30]. All studies were in English, apart from three published in Spanish [27, 32, 34] and two in Portuguese [30, 35].

All studies were cross-sectional in nature (Table 1), the majority (22/25; 88%) were performed from university clinics, and originated from fifteen countries (Brazil, Canada, Colombia, Finland, India, Indonesia, Iran, Jordan, Mexico, Nigeria, Saudi Arabia, South Africa, Sweden, Turkey, and the United States of America). A wide variety of patient samples and corresponding indications for panoramic radiographs were covered, including: patients seeking orthodontic treatment (*n* = 11; 44%), patients seeking dental treatment (*n* = 6; 24%), patients seeking pedodontics treatment or referred to a radiology clinic (*n* = 2; 8% each), or other reasons (patients referred to hospital, public insurance referrals, patients referred for 3rd molar extraction, dental students, and patients with temporomandibular joint symptoms [*n* = 1; 4% each]). As far as ages are concerned, six studies (24%) included adult patients, four studies (16%) included underage (< 18 years) patients, the remaining studies included mixed underage/adult patients, and five studies (20%) did not report age ranges. Among the 14 studies reporting mean age, the median age was 28.9 years (interquartile range 17.2 to 38.5 years). The median study sample size was 526 patients (interquartile range 249 to 1032 patients; range 30 to 43880) for a total of 66 882 patients. Among the 22 studies reporting patient gender, 44.1% of included patients were male (9266/21008 patients). Only eight (32%) of the included studies provided sufficient technical details about the panoramic radiographs, while nine (36%) provided limited information and eight (32%) provided no information. In seven (28%) of the included studies, the panoramic radiograph assessor was reportedly trained or calibrated beforehand, while oral/maxillofacial radiologists were employed in less than half (*n* = 12; 48%) of included studies. Intra- or inter-examiner agreement among multiple assessors was assessed in 11 (44%) of the included studies. In the majority of included studies (*n* = 23; 92%), a wide variety of incidental findings were reported, while two studies (8%) reported only on dental anomalies. In nine (36%) of the studies included, specific findings were not considered incidental and were omitted, with the position of the third molars being the most often one omitted.

**Table 1:** Descriptives of included studies

Study	Design; setting; country^€^	Patients	Age range	Mean age	*n* (male %)	Radiograph details	Assessors	Reliability INTRA/ INTER	Findings
Aikins 2022 [42]	CS; Uni; NGA	Visiting Ortho clinic	5.0–44.0	14.6	249 (43.4%)	NR	2x NR	Kappa 0.95/0.89	Dental anomalies
Al-Abdallah 2015 [28]	CS; Uni; JOR	Seeking Ortho / Pedo Tx	8.6–25.4	17.3	3315 (34.3%)	Digital	2x calibrated (1x Ortho / 1x OMS)	NR	Dental anomalies
Almeida 2024 [44]	CS; Uni; CAN	Public insurance referrals	18.0–84.0	38.5	526 (57.4%)	Planmeca ProOne^®^	1x trained GD; cross-checked by 1x OMR	NR	Various
Bondemark 2006 [4]	CS; Uni; SWE	Visiting Ortho clinic	NR	11.2	496 (53.2%)	Ortophos CD (60 kV; 9–10 mA; 14.1 s) / Cranex 3 + Ceph (65–67 kV; 6 mA; 16–19 s)	2x observers; cross-checked by 1x OMR	Kappa 0.86/0.64	Various (caries, tooth number anomalies, and eruption disturbances ignored)
Büyükgöze-Dindar 2022 [43]	CS; Uni; TUR	Visiting dental clinic	NR*	NR*	43,880 (NR*)	PaX-Flex3D	2x observers	Kappa -/0.89	Various (third molars excluded)
Cral 2016 [30]	CS; Uni; BRA	Visiting Ortho clinic & after Ortho Tx	11.0–18.0	NR	250 (43.6%)	NR	1x trained examiner	Kappa 0.97/-	Various (caries, PDL thickening, and maxillary sinus alterations ignored)
da Silva Dias 2019 [35]	CS; Uni; BRA	Visiting dental clinic	60.0–97.0	67.8	1006 (33.8%)	KODAK^®^ 8000 C digital (74 kV; 8 mA; 13.9 s; 15% magnification)	1x calibrated researcher; supervised by 1x OMR	Kappa 0.88/-	Various
Edmonds 2025^†^ [47]	CS; Uni; CAN	Visiting Ortho clinic	7.0–21.0	13.6	1756 (46.1%)	Morita^®^ Veraview X800 / KODAK^®^ 8000 C / KODAK^®^ 9000	OMR PGs; supervised by 1x senior OMR	-	Various (third molars excluded)
Escolano Rivas 2018 [34]	CS; Clin; COL	Edentulous patients visiting radiology clinics	41.0–91.0	65.8	112 (29.5%)	-	1x OMS PG; supervised by 1x OMR	-	Various
Hernández 2018 [5]	CS; Uni; COL	Visiting Ortho clinic	3.0–70.0	23.3	783 (42.1%)	Ortophos XG plus DS/Ceph (60–77 kV; 8–15 mA; 9.4–14.1 s)	1x oral pathology expert	Kappa 0.64/-; BA-LOA − 0.24,0.04	Various
Hlongwa 2023 [3]	CS; Uni; ZAF	Visiting Ortho clinic	7.0–57.0	17.2	100 (41.0%)	Orthophos XG3D/Ceph (60–90 kV; 3–16 mA; 9.4–14.1 s)	NR	Kappa 0.85/0.81	Various (caries, tooth number anomalies, crowding, and eruption disturbances not recorded as IFs)
Imanimoghaddam 2021 [40]	CS; Uni; IRN	Visiting dental clinic	4.0–84.0	34.4	1987 (40.0%)	Rayscan digital	2x calibrated OMR	-	Various
Indra 2020 [36]	CS; Uni; IND	Patients who had Tx in an Ortho clinic	NR	NR	30 (40.0%)	NR	2x examiners	Kappa − 0.85/0.60	Various
Jadu 2015 [29]	CS; Uni; SAU	Referred for third molar extraction	14.0–77.0	36.5	121 (50.4%)	Care stream 8000 digital	1x OMR	-	Various
Jiménez Ortíz 2017 [32]	CS; Uni; MEX	Dental students	19.0–44.0	19.5	98 (29.6%)	Digital	2x assessors	-	Various
Karkac 2024 [45]	CS; Prac; TUR	Seeking Ortho Tx in private practices	6.0–49.0	NR	330 (48.5%)	NR	NR	-	Various
Leyva Altamirano 2011 [27]	CS; Uni; MEX	Visiting Ortho clinic	10.0-	NR	603 (37.8%)	NR	NR assessors; IFs confirmed by 1x OMR	-	Various
MacDonald 2020 [37]	CS; Uni; CAN	Seeking dental check-up or dental hygiene	7.0–90.0	NR	6252 (50.7%)	Planmeca PM 2002 CC Proline	NR	-	Various (implants, caries, endodontics, restorations, and impacted third molars not recorded as IFs)
Mohan 2024 [46]	CS; Uni; USA	Visiting dental clinic	6.0–18.0	NR	581 (57.1%)	Planmeca ProMax / Sirona ORTHOPHOS XG (60–78 kV; 8–16 mA; 9.5–14.0 s)	1x assessor, calibrated & confirmed by 1x OMR	Kappa -/0.90	Various
Pakbaznejad Esmaeili 2016 [31]	CS; Uni; FIN	Mostly (92–95%) seeking Ortho Tx^¥^	7.0–12.0	NR	413 (49.2%)	NR	1x assessor, confirmed by 1x OMR	Kappa -/0.53-1.00	Various (third molars excluded)
Pamadya 2025 [48]	CS; Hosp; IDN	Visiting a hospital	NR	NR	962 (NR)	NR	2x calibrated assessors	-	Various
Sadik 2020 [38]	CS; Uni; TUR	Edentulous patients visiting dental clinic	35.0–87.0	64.6	508 (40.2%)	Kodak 8000 C digital	2x Perio with 1x OMR	-	Various
Temur 2021 [41]	CS; Uni; TUR	Patients visiting a Pedo clinic	NR-18.0	NR	1432 (40.2%)	GENDEX GDP − 700 (66 kV; 6.3 mA; 14 s)	1xOMR	-	Various (third molars excluded)
Vaiciulis 2020 [39]	CS; Uni; BRA	Patients with painful TMDs	18.0-	36.9	60 (36.7%)	Panex EC Veraview (70–90 kV; 10 mA)	NR	-	Various
Yoga 2017 [33]	CS; Uni; IND	Referred to radiology department	NR	NR	1032 (NR)	Orthophos XG digital (80 kV; 12 mA)	-	Kappa 0.95/-	Various (dental extractions, third molar impactions, maxillary sinus thickening, and caries were not recorded as IFs)

*BA-LOA* Bland-Altman limits of agreement, *Clin* clinic, *CS* cross-sectional study, *GD* general dentist, *Hosp* hospital, *NR* not reported; *OMR* oral & maxillofacial radiologist, *OMS* oral & maxillofacial surgery specialist, *Ortho* orthodontic / orthodontist, *PDL* periodontal ligament, *Pedo* pediatric dentistry/dentist, *Perio* periodontist, *IF* incidental finding, *PG* post-graduate residents, *Prac* private practice, *TMD* temporomandibular disorder, *Tx* treatment, *Uni* university clinic

^€^ countries give with their ISO 3166-1 alpha-3 codes

* demographics given for the subsample of patients with at least one dental anomaly, not the parent sample assessed

^†^ study included a sub-study where 12 selected panoramic radiographs were collected and administered on a survey of orthodontists; this was excluded from this review

^¥^ including patients with general health problems (3.6%)

### Risk of bias/issues with the internal validity of included studies

**Fig. 2:**
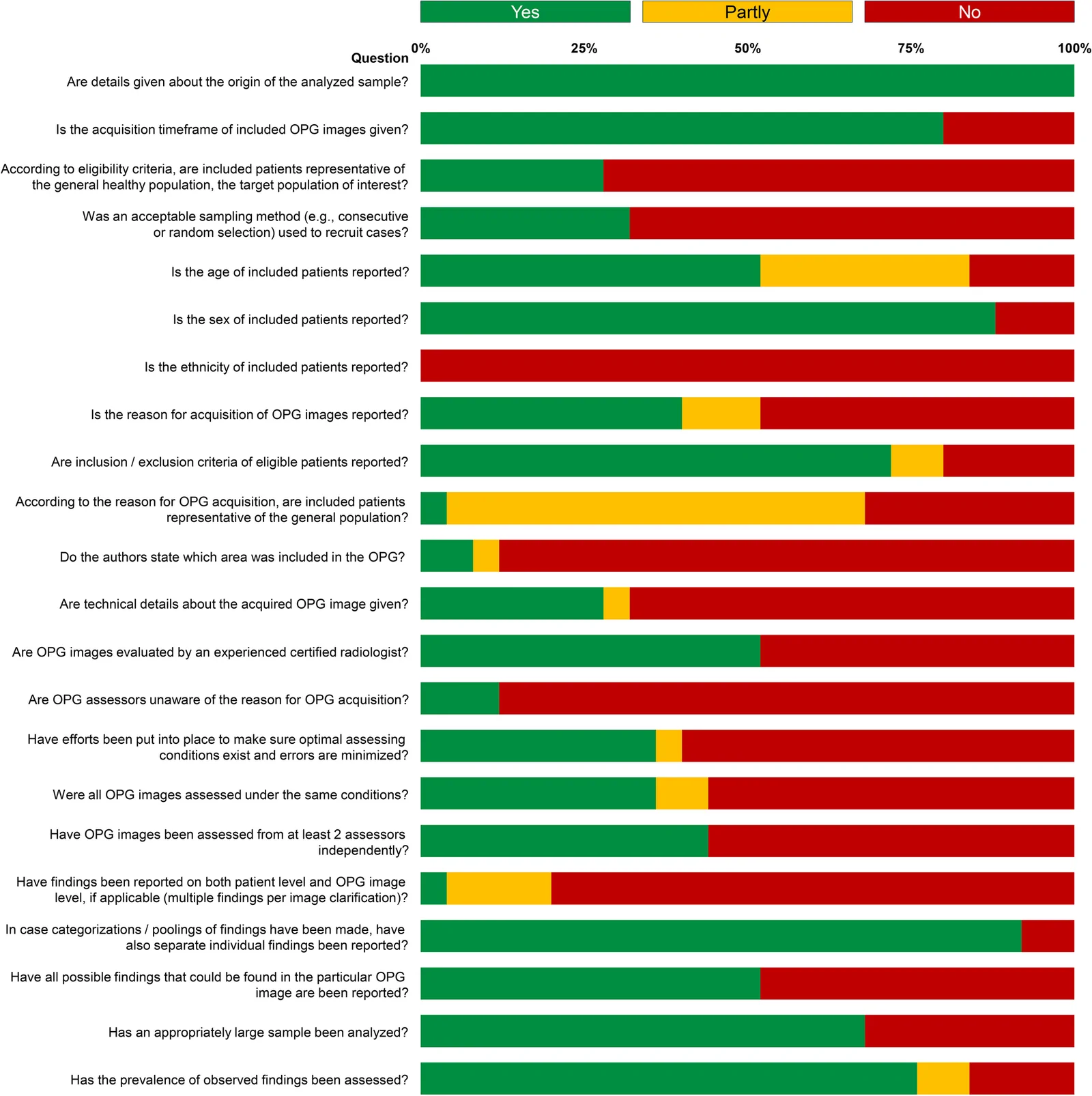
Summary of the risk of bias / methodological issues of included studies

The assessment of methodological characteristics and internal validity factors of included studies can be seen in the summary in Table 2/Fig. 2 and in full detail in Appendix 6. Most problematic domains were non-reported or incomplete reporting of the patients’ ethnicity (100%), detail of the panoramic radiograph (88% of the cases), assessor not being blinded to the acquisition reason for the panoramic radiograph (88%), non-reporting of finding at both patient and panoramic radiograph level (i.e. if multiple finding were detected per patient) (80%), lack of information about the representativeness of the included sample (72%), lack of information about the recruitment and selection of the included sample (68%), lack of information about the optimal assessment conditions to minimize errors (60%), lack of duplicate assessment of included panoramic radiograph (56%), lack of assessment by an experienced certified radiologist (48%), and lack of an appropriately large sample size (32%).

**Table 2:** Summary of the risk of bias / methodological issues of included studies

Nr	Question	Yes *n* (%)	Partly *n* (%)	No *n* (%)
1	Are details given about the origin of the analyzed sample?	25 (100%)	-	-
2	Is the acquisition timeframe of included panoramic radiographs given?	20 (80%)	-	5 (20%)
3	According to eligibility criteria, are included patients representative of the general healthy population, the target population of interest?	7 (28%)	-	18 (72%)
4	Was an acceptable sampling method (e.g., consecutive or random selection) used to recruit cases?”	8 (32%)	-	17 (68%)
5	Is the age of included patients reported?	13 (52%)	8 (32%)	4 (16%)
6	Is the sex of included patients reported?	22 (88%)	-	3 (12%)
7	Is the ethnicity of included patients reported?	-	-	25 (100%)
8	Is the reason for acquisition of panoramic radiographs reported?	10 (40%)	3 (12%)	12 (48%)
9	Are inclusion / exclusion criteria of eligible patients reported?	18 (72%)	2 (8%)	5 (20%)
10	According to the reason for panoramic radiograph acquisition, are included patients representative of the general population?	1 (4%)	16 (64%)	8 (32%)
11	Do the authors state which area was included in the panoramic radiograph?	2 (8%)	1 (4%)	22 (88%)
12	Are technical details about the acquired panoramic radiograph given?	7 (28%)	1 (4%)	17 (68%)
13	Are panoramic radiographs evaluated by an experienced certified radiologist?	13 (52%)	-	12 (48%)
14	Are assessors unaware of the reason for panoramic radiograph acquisition?	3 (12%)	-	22 (88%)
15	Have efforts been put into place to make sure optimal assessing conditions exist and errors are minimized?	9 (36%)	1 (4%)	15 (60%)
16	Were all panoramic radiographs assessed under the same conditions?	9 (36%)	2 (8%)	14 (56%)
17	Have panoramic radiographs been assessed from at least two assessors independently?	11 (44%)	-	14 (56%)
18	Have findings been reported on both patient level and panoramic radiograph image level, if applicable (multiple findings per image clarification)?	1 (4%)	4 (16%)	20 (80%)
19	In case categorizations / poolings of findings have been made, have also separate individual findings been reported?	23 (92%)	-	2 (8%)
20	Have all possible findings that could be found in the particular panoramic radiograph image been reported?	13 (52%)	-	12 (48%)
21	Has an appropriately large sample been analyzed?	17 (68%)	-	8 (32%)
22	Has the prevalence of observed findings been assessed?	19 (76%)	2 (8%)	4 (16%)

### Results of individual studies

The results of the individual included studies can be found in the review’s openly available dataset [26]. The prevalences of incidental findings assessed by at least two studies were pooled through meta-analyses, while findings assessed by single studies were reported separately in Appendix 7. These included periodontal pathology (3.8%), mesiodens (3.6%), third molar inside the mandibular canal (3.3%), endodontic treatment (3.2%), and root tip embedded in bone (1.4%).

### Data synthesis

**Fig. 3:**
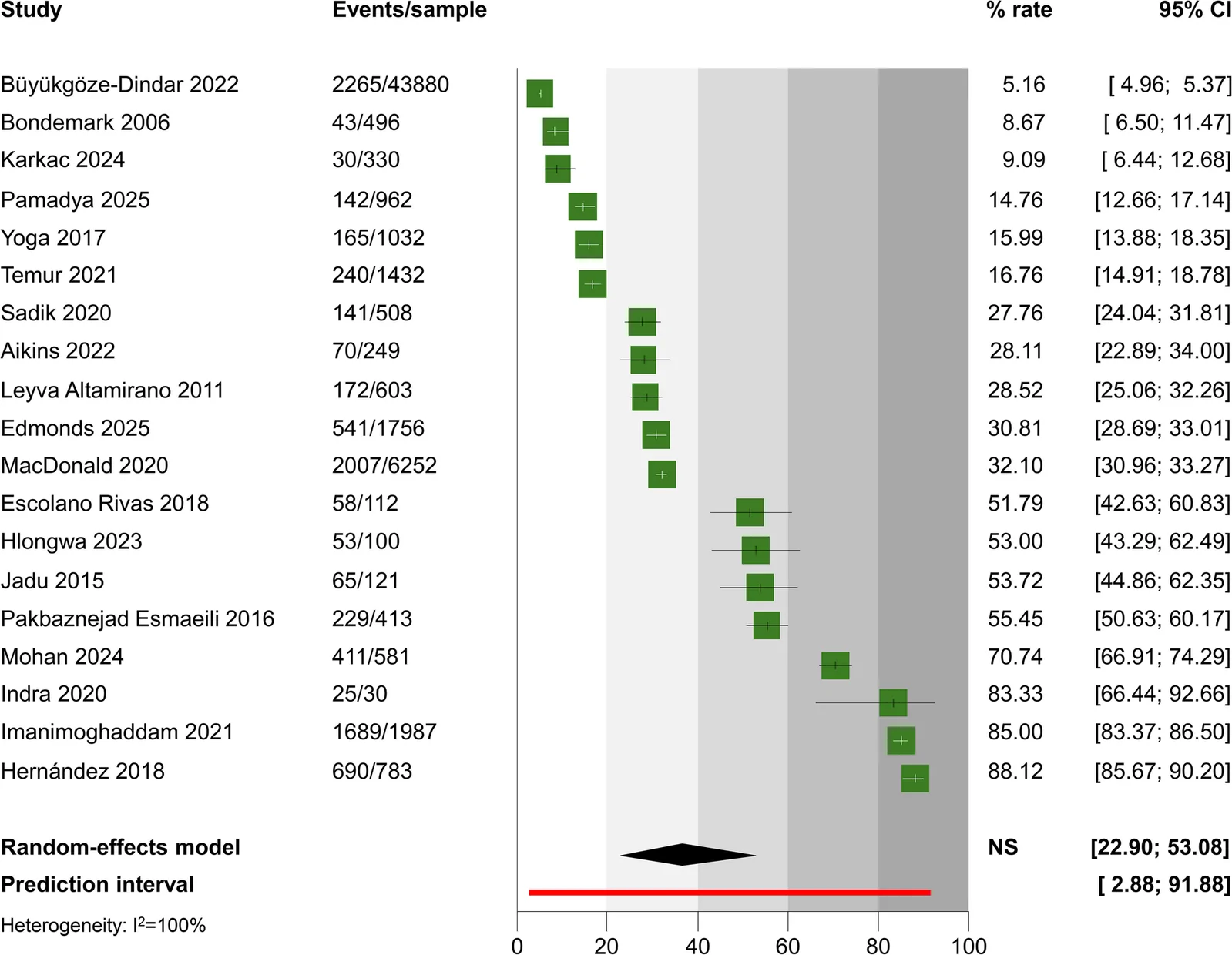
Forest plot for the pooled prevalence of having at least one incidental finding. OPG, orthopantomogram

Initially, the prevalence of patients with at least one incidental finding was pooled across studies that performed comprehensive assessments of acquired panoramic radiographs. It is important to state here that studies with patients not representative of the average patient — i.e., patients referred for temporomandibular joint problems —were excluded from the main analysis. A meta-analysis of 19 studies and 61,627 patients (Table 3; Fig. 3) indicated substantial between-study variability, as reflected by both absolute and relative heterogeneity. As such, even though the pooled prevalence was 36.7% (95% CI 22.9%-53.1%), estimating the point estimate may be problematic (it may be imprecise), and the 95% CI would be more informative.

**Table 3:** Results of performed meta-analyses

No	Incidental finding	Studies (patients)	Pooled %rate (95% CI)	I^2^ / tau^2^	95% prediction
	Any finding (no category)	19 (61627)	36.70 (22.90, 53.08)	100% / 1.90	2.88, 91.88
1. Dental Developmental & Morphologic Findings					
1	Amelogenesis imperfecta	3 (46650)	0.13 (0.02, 0.75)	96% / 2.26	0, 70.75
2	Dens invaginatus	3 (47623)	0.11 (0.02, 0.54)	96% / 1.86	0, 50.39
3	Enamel pearl	5 (4897)	0.85 (0.17, 4.10)	89% / 1.30	0.02, 22.92
4	Fusion/gemination	3 (47623)	0.03 (0, 0.17)	87% / 2.04	0, 30.21
5	Hyperdontia	13 (58836)	1.34 (0.90, 2.00)	94% / 0.33	0.36, 4.81
6	Hypodontia	11 (59943)	4.45 (2.37, 8.21)	99% / 0.91	0.50, 30.04
7	Impaction	11 (58650)	10.53 (3.56, 27.29)	100% / 2.94	0.22, 86.46
8	Macrodontia	3 (47299)	0.07 (0, 10.41)	95% / 3.97	0, 93.69
9	Microdontia	7 (49034)	1.10 (0.67, 1.80)	85% / 0.15	0.37, 3.17
10	Oligodontia	2 (45636)	0.04 (0.03, 0.07)	47% / 0	0, 0.75
11	Retained tooth	2 (350)	3.39 (1.30, 8.55)	52% / 0.01	0.01, 95.66
12	Root dilaceration	6 (8885)	1.51 (0.31, 6.99)	98% / 1.99	0.03, 44.52
13	Summary: dental findings	5 (9642)	12.19 (1.54, 55.23)	98% / 3.02	0.07, 96.53
14	Summary: tooth number anomaly	2 (44461)	6.13 (1.38, 23.37)	100% / 1.23	0, 100.00
15	Summary: tooth position anomaly	3 (44874)	11.68 (3.49, 32.56)	100% / 1.30	0.05, 97.47
16	Summary: tooth shape anomaly	5 (45470)	2.85 (0.39, 18.06)	99% / 2.47	0.02, 78.22
17	Talon cusp	3 (46448)	0.17 (0.03, 1.05)	98% / 2.33	0, 79.08
18	Taurodontism	7 (50668)	1.00 (0.21, 4.63)	99% / 2.79	0.01, 44.59
19	Tooth ectopia	5 (48495)	0.25 (0.09, 0.70)	83% / 0.42	0.03, 1.95
20	Transposition	5 (48453)	0.15 (0.03, 0.81)	91% / 1.39	0, 5.70
2. Odontogenic & Periapical Pathology					
21	Cementoblastoma	2 (1386)	0.43 (0.19, 0.96)	34% / 0	0, 44.04
22	Cemento-osseous dysplasia (periapical)	4 (3263)	0.57 (0.02, 13.69)	95% / 3.65	0, 85.42
23	Cyst; bone	2 (1856)	0.51 (0.11, 2.27)	82% / 0.41	0, 99.94
24	Cyst; dentigerous	7 (10240)	0.73 (0.16, 3.25)	98% / 2.27	0.01, 28.45
25	Cyst; radicular	3 (2480)	0.41 (0.07, 2.58)	82% / 1.99	0, 86.05
26	Dense bone island / osteosclerosis	9 (12832)	3.91 (1.70, 8.73)	93% / 1.64	0.18, 47.94
27	Hypercementosis	2 (1033)	1.43 (0.35, 5.69)	74% / 0.60	0, 99.99
28	Odontoma	6 (4966)	0.42 (0.20, 0.87)	50% / 0.13	0.13, 1.35
29	Osseous lesion	2 (1022)	2.19 (0.19, 20.62)	95% / 2.87	0, 100.00
30	Osteitis condensans / sclerosing osteitis	2 (7035)	1.64 (0.82, 3.23)	94% / 0.22	0, 96.63
31	Periapical lesion / rarefying osteitis	9 (6618)	5.32 (1.37, 18.48)	99% / 3.17	0.07, 81.07
32	Radiolucency	3 (6953)	3.65 (0.20, 41.77)	98% / 1.35	0.01, 92.57
33	Radiopacity	5 (8030)	7.14 (3.22, 15.11)	97% / 0.40	1.08 35.19
34	Retained root	3 (3288)	5.27 (0.33, 48.00)	98% / 6.04	0, 99.99
35	Root resorption	7 (12581)	0.85 (0.39, 1.83)	88% / 0.54	0.12, 5.75
3. Calcifications & Soft-Tissue Findings					
36	Calcification; antrolith	2 (3743)	0.06 (0.01, 0.58)	0% / 0.81	0, 100.00
37	Calcification; carotid artery	2 (1558)	1.00 (0.18, 5.48)	93% / 1.40	0, 100.00
38	Calcification; pulp	3 (1133)	3.24 (0.71, 13.54)	78% / 0.26	0.23, 32.68
39	Calcification; sialolith	4 (10116)	0.20 (0.03, 1.39)	83% / 1.06	0, 8.31
40	Calcification; styloid ligament (/elongation)	8 (7472)	7.12 (0.99, 36.94)	99% / 5.79	0.02, 96.99
41	Calcification; tonsilolith	4 (10116)	2.68 (0.98, 7.12)	95% / 0.33	0.33, 18.46
42	Calcification; vascular plaque	2 (2108)	3.65 (2.93, 4.54)	0% / 0	0.86, 14.22
43	Foreign body	2 (904)	1.02 (0.22, 4.56)	88% / 0.91	0, 100.00
44	Summary: soft-tissue calcifications	3 (3070)	9.41 (4.14, 19.99)	98% / 0.56	0.24, 81.52
4. Maxillary Sinus & Nasal Findings					
45	Nasal septum deviation	2 (1854)	2.74 (0.01, 91.01)	98% / 17.40	0, 100.00
46	Sinus; hypoplasia	2 (3743)	0.19 (0.09, 0.39)	0% / 0	0, 18.65
47	Sinus; inflammation	2 (2539)	0.52 (0.09, 3.07)	83% / 1.23	0, 100.00
48	Sinus; mucosal thickening	5 (3307)	2.54 (0.78, 7.99)	92% / 0.71	0.19, 26.49
49	Sinus; mucous retention (pseudo)cyst	6 (11502)	1.87 (0.48, 6.93)	97% / 1.55	0.06, 38.05
50	Sinus; opacity	2 (2108)	1.57 (1.12, 2.19)	0% / 0	0.17, 12.88
51	Sinus; pneumatization	3 (1887)	50.64 (32.63, 68.49)	99% / 0.42	3.84, 96.35
52	Summary: airway findings	3 (3120)	30.86 (2.16, 90.03)	100% / 7.04	0, 100.00
53	Summary: sinus findings	4 (4826)	8.22 (3.06, 20.25)	98% / 0.40	0.92, 46.27
54	Turbinate hypertrophy	3 (2637)	6.98 (0.13, 81.75)	98% / 12.70	0, 100.00
5. TMJ Findings					
55	Condyle; degenerative joint disease	2 (1877)	0.11 (0.03, 0.43)	72% / 0	0, 89.53
56	Condyle; hyperplasia	2 (2539)	0.32 (0.16, 0.63)	24% / 0	0, 22.14
57	Summary: TMJ finding	6 (4876)	1.77 (0.50, 6.15)	98% / 1.35	0.07, 31.84
6. Skeletal / Postoperative / Miscellaneous Findings					
58	Marginal bone loss	6 (4667)	6.13 (0.11, 79.58)	100% / 14.73	0, 99.96
59	Osteosynthesis material	2 (6502)	1.05 (0.83, 1.32)	0% / 0	0.22, 4.74
60	Summary: vertebrae findings	2 (1364)	7.41 (0.34, 65.11)	99% / 5.06	0, 100.00

*CI* confidence interval, *TMJ* temporomandibular joint

Subgroup analyses were performed to try to explain sources of heterogeneity in the pooled prevalence of incidental findings (Table 4). Analysis according to the sample analyzed in each study indicated that statistically significant differences existed in the prevalence of incidental findings among patients seeking pedodontic treatment (16.8%), patients visiting a public health clinic (33.6%), patients seeking orthodontic treatment (39.0%), patients seeking general dental treatment (50.4%), and patients referred for 3rd molar extraction (53.7%). The prevalence of incidental findings was 38.5% (95% CI 23.5%-56.1%) among edentulous patients and 88.3% (95% CI 77.5%-94.3%) among patients referred for temporomandibular joint disorders, but these were excluded from the main analysis. Even though adult patients had a higher prevalence of incidental findings than children (83.1% versus 52.7%), this did not reach the 10% threshold for statistically significant between-subgroup differences (*P* = 0.13). Meta-regression analyses did not find significant effects of mean age or % of male patients in the study sample (*p* > 0.10 in both instances; Table 5).

**Table 4:** Subgroup analysis for the prevalence of any incidental finding

Factor	Subgroup	Studies (patients)	Pooled %rate (95% CI)	I^2^ / tau^2^	*p*_SG_
Sample origin	Seeking pedodontic treatment	1 (1432)	16.76 (14.91, 18.78)	- / -	< 0.001
	Seeking orthodontic treatment	9 (4980)	38.98 (20.88, 60.73)	99% / 1.81	
	Seeking dental treatment	6 (53732)	50.37 (17.27, 83.14)	100% / 3.90	
	Visiting public health clinic	2 (1488)	33.60 (10.24, 69.18)	100% / 1.15	
	3rd molar extraction	1 (121)	53.72 (44.81, 62.40)	- / -	
Patient age group	Children	4 (2676)	52.71 (28.80, 75.43)	100% / 1.06	0.13
	Adults	2 (1532)	83.11 (48.12, 96.31)	100% / 1.44	

*CI* confidence interval, *p*_*SG*_ p value for between-subgroups differences

**Table 5:** Meta-regression analyses of incidental findings by mean age and % of male patients in the sample

Factor	Studies	Comparison	Coefficient (95% CI)	*p*
Age	9	Per + 10 years	0.10 (-0.41, 0.61)	0.65
Male %	16	Per + 10% male patients	-0.46 (-1.50, 0.58)	0.36

*CI* confidence interval

The 61 separate findings reported from the acquired panoramic radiographs were categorized into six categories: (1) dental developmental & morphologic findings; (2) odontogenic & periapical pathology; (3) calcifications & soft-tissue findings; (4) maxillary sinus & nasal findings; (5) temporomandibular joint findings; and (6) skeletal/postoperative/miscellaneous findings.

As far as dental developmental & morphologic findings are concerned, mostly anomalies of tooth position were found (11.7%), followed by anomalies of tooth number (6.1%), and anomalies of tooth shape (2.9%). Among specific findings, tooth impaction had the highest prevalence (10.5%), followed by hypodontia (4.5%), tooth retention (3.4%), hyperdontia (1.3%), microdontia (1.1%), and taurodontism (1.0%).

Odontogenic and periapical pathology was found in several patients, which included findings like radiopacities (7.1%), periapical lesions / rarefying osteitis (5.3%), retained roots (5.3%), dense bone islands/osteoclerosis (3.9%), radiolucencies (3.7%), sclerosing osteitis (1.6%), and hypercementosis (1.4%).

Soft-tissue calcifications were found with an average pooled prevalence of 9.4% and included various findings. The most common findings were calcification/elongation of the styloid ligament (7.1%), followed by vascular plaques (3.7%), pulp calcifications (3.2%), tonsiloliths (2.7%), and calcifications of the carotid artery (1.0%).

Maxillary sinus and nasal findings were very often reported across the included studies, with airway findings in 30.9% of patients and sinus findings in 8.2%. By far the most commonly reported finding was pneumatization of the sinus (50.6%), followed by turbinate hypertrophy (7.0%), nasal septum deviation (2.7%), and mucosal thickening of the sinus (2.5%).

Temporomandibular joint findings were observed in 1.8% of patients, with the most commonly reported findings being condylar hyperplasia (0.3%) and degenerative joint disease (0.1%).

Finally, other miscellaneous findings reported included vertebral anomalies (7.4%) and marginal bone loss (6.1%).

### Additional analyses

The presence and impact of reporting bias (including the possibility of small-study effects and publication bias) were assessed using a contour-enhanced funnel plot (Appendix 8). Visual inspection of the funnel plot indicated no asymmetry, which was confirmed by the Thompson-Sharp test (*p* = 0.14).

Nevertheless, a sensitivity analysis was conducted to assess the potential for small-study effects or imprecision due to the small sample sizes of the included studies. Analyzing study subsets by sample size (Appendix 9) indicated that studies with larger samples tended to show lower rates of incidental findings than those with smaller samples. Therefore, based on the expected prevalence of each incidental finding, future studies should ensure appropriate sample sizes to achieve adequate statistical power.

## Discussion

This systematic review and meta-analysis synthesized evidence from 25 cross-sectional studies comprising more than 61,000 patients and demonstrated that incidental findings are a common feature of dental panoramic radiography. The pooled prevalence of patients presenting with at least one incidental finding was 36.7% (95% CI: 22.9%–53.1%), indicating that more than one-third of patients undergoing panoramic imaging harbor radiographic findings unrelated to the primary indication for imaging. This observation is consistent with previous studies reporting a high frequency of incidental findings and emphasizing the need for systematic evaluation of all anatomical structures captured within the radiographic field rather than restricting interpretation to the immediate area of clinical interest [3, 4, 10, 49]. The prevalence observed in the present review is broadly comparable to estimates reported in individual studies, in which incidental findings have been identified in approximately one-quarter to one-half of patients examined [3, 10, 15]. However, the substantial heterogeneity observed between studies suggests that differences in patient demographics, referral pathways, healthcare settings, examiner characteristics, and the criteria used to define and record incidental findings influence prevalence [5, 7]. Therefore, although the pooled estimate should be interpreted cautiously, the available evidence clearly indicates that incidental findings are routinely encountered in clinical practice and should not be regarded as exceptional observations.

The marked heterogeneity observed across studies appears to be both methodological and clinical in origin. Included studies varied considerably in patient populations, referral indications, healthcare settings, definitions of incidental findings, and the extent of radiographic assessment. Several studies excluded specific categories of findings, particularly third molar-related abnormalities, whereas others focused on selected pathological entities or developmental anomalies. Furthermore, methodological shortcomings were common, including incomplete reporting of imaging protocols, lack of examiner calibration, inconsistent involvement of oral and maxillofacial radiologists, and variation in image interpretation procedures [1, 2, 4, 6, 49]. These factors likely contributed substantially to variability in reported prevalence estimates. Subgroup analyses further demonstrated significant differences across patient populations, with prevalence increasing from pediatric to orthodontic, general dental, and individuals referred for third molar extraction. This pattern is biologically plausible and likely reflects the cumulative burden of developmental, inflammatory, and degenerative changes that increase with age and disease exposure [8, 9, 15, 50]. Although age was not a significant predictor in meta-regression analyses, adult populations showed consistently higher prevalence estimates than younger cohorts, consistent with previous reports from both panoramic and three-dimensional imaging studies [10, 51, 52]. Conversely, the lower prevalence observed in paediatric and orthodontic populations likely reflects younger baseline ages and reduced cumulative exposure to acquired pathological processes [5].

Dental developmental, morphological, and intraosseous findings were among the largest categories of incidental findings identified in this review. Anomalies of tooth position, particularly impacted teeth, together with anomalies of tooth number such as hypodontia, accounted for a substantial proportion of all detected abnormalities. These findings are consistent with previous studies demonstrating the value of panoramic radiography for identifying disturbances in tooth development and eruption, particularly in orthodontic populations where such abnormalities may significantly influence treatment planning and prognosis [3–5, 9]. The relatively high prevalence of impacted teeth likely reflects the inclusion of orthodontic patients and individuals referred for third molar assessment, both of which are enriched for eruption-related abnormalities. In addition, odontogenic and periapical pathologies constituted a considerable proportion of incidental findings, including radiopacities, dense bone islands/osteosclerosis, retained roots, periapical lesions, and radiolucencies. The prevalence of retained roots and periapical radiolucencies is particularly noteworthy, as these findings may represent asymptomatic, previously undiagnosed endodontic disease [15, 50]. Although many of these abnormalities are clinically silent, they may influence restorative, implant, orthodontic, or surgical treatment planning if left unidentified. Collectively, these findings highlight the ability of panoramic radiography to reveal both developmental anomalies and occult pathological processes that may not be evident during routine clinical examination [3–5, 15].

Soft-tissue calcifications constituted another clinically relevant category of incidental findings, with a pooled prevalence of 9.4%. The most common findings included stylohyoid ligament calcification, vascular calcifications, carotid artery plaques, tonsilloliths, and pulp calcifications. While many of these findings are asymptomatic, some may have important diagnostic implications. Stylohyoid ligament mineralization is generally considered an anatomical variation but may occasionally be associated with Eagle syndrome and should therefore be considered in the differential diagnosis of unexplained cervicofacial symptoms [5, 15]. Of greater systemic significance is the detection of carotid artery calcifications, which have been associated with atherosclerotic disease and an increased risk of future cardiovascular and cerebrovascular events. Because panoramic radiographs routinely include the carotid bifurcation region, they may facilitate the incidental detection of calcified atherosclerotic plaques [10]. Although panoramic radiography cannot determine the presence or severity of carotid stenosis and should not be considered a diagnostic tool for vascular disease, several studies have demonstrated that such findings may serve as markers of elevated cardiovascular risk and justify further medical assessment in selected patients [11–14]. These observations further illustrate the broader relevance of panoramic radiography in healthcare and underscore the importance of recognizing soft-tissue calcifications during routine image interpretation [7, 11, 51].

Maxillary sinus, nasal, and airway-related findings also represented a substantial proportion of incidental abnormalities. Airway findings were identified in nearly one-third of patients, while sinus abnormalities were present in 8.2% of cases. Sinus pneumatization exhibited a particularly high prevalence, whereas turbinate hypertrophy, nasal septum deviation, and mucosal thickening were less frequently reported. The predominance of sinus pneumatization likely reflects differences in how studies classified anatomical variations and pathological findings. The frequency of sinonasal findings is unsurprising given the close anatomical relationship between the dentition, maxillary sinus, nasal cavity, and upper airway, all of which are included within the panoramic field of view [3, 5]. Clinically, the identification of sinus pneumatization and mucosal abnormalities may be particularly relevant when planning implant placement, sinus augmentation procedures, or orthognathic surgery [10, 52]. However, the diagnostic capabilities of panoramic radiography remain limited by distortion, anatomical superimposition, and its two-dimensional nature. Studies comparing panoramic radiography with computed tomography and cone-beam computed tomography have demonstrated that some sinus abnormalities may be underestimated or incompletely characterized on panoramic images alone [4, 51, 53, 54]. Accordingly, suspicious findings often require further three-dimensional imaging or specialist evaluation. The identification of vertebral anomalies and marginal bone loss further illustrates the broad anatomical coverage of panoramic radiography and its ability to reveal findings beyond the dentoalveolar region [3, 5].

Several limitations of the available evidence should be considered when interpreting these findings. Despite providing the most comprehensive synthesis currently available, the review identified widespread deficiencies in study design and reporting. Most studies provided limited information regarding imaging parameters, assessor blinding, examiner calibration, and participant selection, while none reported patient ethnicity [1, 2, 6, 7]. Furthermore, many studies failed to distinguish between patient-level and radiograph-level findings, limiting comparisons across investigations and potentially affecting prevalence estimates [50]. Fewer than half of the included studies employed certified oral and maxillofacial radiologists, and only a minority reported formal calibration procedures or assessment reliability, factors that may have contributed substantially to the observed heterogeneity [3, 5]. Future research would benefit from standardized definitions of incidental findings, calibrated and blinded image assessment, detailed reporting of imaging protocols, and consistent reporting at both patient and radiograph levels [5, 7, 50]. Longitudinal studies are also needed to determine the clinical significance, referral outcomes, and cost-effectiveness of managing incidental findings detected on panoramic radiographs. In parallel, emerging artificial intelligence systems may improve consistency and efficiency in image interpretation, although their diagnostic performance and clinical integration require further validation [1, 2, 6, 55]. Overall, the high prevalence and broad spectrum of incidental findings identified in this review reinforce the need for systematic evaluation of all anatomical structures visible on panoramic radiographs, irrespective of the original indication for imaging [3, 10].

## Conclusions

Incidental findings are common on dental panoramic radiographs, affecting more than one-third of patients, with considerable variation across clinical populations and study settings. Developmental dental anomalies, odontogenic pathologies, soft-tissue calcifications, and sinonasal findings were among the most frequently identified abnormalities. Although many incidental findings are clinically insignificant, some may have important implications for dental treatment planning or broader patient management. These findings underscore the need for systematic evaluation of all anatomical structures visible on panoramic radiographs and highlight the importance of standardized methodologies in future prevalence research.

## Supplementary Information

Below is the link to the electronic supplementary material.

Supplementary Material 1

## Data Availability

Data availability All data have been made available through Zenodo (doi: 10.5281/zenodo.20522989).
