# Supplementary material for: Incidental findings from panoramic radiographs: A systematic review with meta-analysis
Source: Clin Oral Investig. 2026 Sep 24;30(10):464. doi: 10.1007/s00784-026-07148-4 (PMC13612610; doi:10.1007/s00784-026-07148-4)
Supplement: Supplementary file 1 [file 784_2026_7148_MOESM1_ESM.pdf]

Incidental findings from panoramic radiographs: a systematic review with meta-analysis

SUPPLEMENTARY MATERIAL

Appendix 1. Additional review details and deviations from the registered protocol.

Additional review details

- Forest plots were enhanced with contours denoting effect magnitude to assess treatment effect magnitude, heterogeneity, and precision (Papageorgiou, 2014). Cut-offs are arbitrary and correspond to 0% and 100% being divided in increments of 20%.
- For all league tables, mixed (direct & indirect) evidence is presented in the lower left part, direct evidence is presented in the upper right part, and statistically significant effects are highlighted in red.

Deviations from registered protocol

- Initially, the review was focused solely on children. However, it was deemed this would lead to it having a very narrow scope (especially since few studies include only children) and it was expanded to dental patients of any age.
- According to this change, the review team was also expanded to include an additional author.
- The initial data synthesis methodology (Freeman-Tukey double arcsine transformation to calculate the 95% confidence intervals of single studies (Lin and Xu 2020). Pooled synthesis will be done with a random-effects (REML) model, with Hartung-Knapp correction, uncertainty around tau2/I2, and random-effects predictions) was modified to a more advanced / conservative approach (A one-step logistic-normal generalized linear mixed model with a logit transformation was chosen, using Wilson score confidence intervals, maximum likelihood estimation for between-study variance, and Hartung-Knapp adjustment), due to its improved performance.
- Several subgroup analyses were initially planned but could not be ultimately conducted due to limited data provision.
- For assessing funnel plot asymmetry, initially the Egger test was chosen. However, this was changed to the Thompson-Sharp test, due to its better performance.
- A post hoc sensitivity analysis was performed according to the sample size of each included study using arbitrary cut-offs: 0-200 patients, 201-500 patients, 501-1000 patients, 1001-2000 patients, >2000 patients.

Appendix 2. Eligibility criteria for study inclusion according to the Participants – Exposure – Comparison – Outcome – Study design (PECOS) framework.

**Component**	**Inclusion**	**Exclusion**
Participants	Patients of any age, sex, or ethnicity with any kind of anamnestic or clinical justification deemed in need to receive a panoramic radiograph for diagnostic imaging reasons	-
Exposure	Panoramic radiograph for diagnostic imaging of the head / neck region	▪ Any kind of imaging modality except panoramic radiograph<br>▪ Any kind of radiography used for therapeutic reasons (like cancer)<br>▪ (Partial) panoramic radiograph of selected / limited area
Comparison	Comparisons according to patient chracteristics	
Outcome	Any incidental finding from panoramic radiograph	Focus on one specific finding solely
Study design	Longitudinal or cross-sectional studies on humans	Animal studies, case series/reports, and non-clinical studies (i.e. cadaver studies)

Appendix 3. Databases searched together with the search strategies and retrieved hits.

Database	Search	Limit	Hits
MEDLINE (via PubMed)	("panoramic" OR "panorex" OR "orthopantomogram" OR orthopantomograp*) AND ("incidental" OR "unexpected" OR "unanticipated" OR "unforeseen") AND ("finding" OR "pathology" OR "lesion" OR "abnormality") AND (dental OR dentistry OR tooth OR teeth OR jaw OR jaws)	Human	139
Scopus	Same as MEDLINE	Human; Dentistry	123
Web of Science	Same as MEDLINE	Dentistry Oral Surgery Medicine	104
Embase	Same as MEDLINE	Human	77
Virtual Health Library	Same as MEDLINE		51

Appendix 4. Custom tool used to assess risk of bias and methodological issues of included studies.

Nr	Question
1	Are details given about the origin of the analyzed sample?
2	Is the acquisition timeframe of included panoramic radiographs given?
3	According to eligibility criteria, are included patients representative of the general healthy population, the target population of interest?
4	Was an acceptable sampling method (e.g., consecutive or random selection) used to recruit cases?"
5	Is the age of included patients reported?
6	Is the sex of included patients reported?
7	Is the ethnicity of included patients reported?
8	Is the reason for acquisition of panoramic radiographs reported?
9	Are inclusion / exclusion criteria of eligible patients reported?
10	According to the reason for panoramic radiographs acquisition, are included patients representative of the general population?
11	Do the authors state which area was included in the panoramic radiograph?
12	Are technical details about the acquired panoramic radiograph given? (kV, mA, exposure time, etc)
13	Are panoramic radiographs evaluated by an experienced certified radiologist?
14	Are panoramic radiograph assessors unaware of the reason for radiograph acquisition?
15	Have efforts been put into place to make sure optimal assessing conditions exist and errors are minimized?
16	Were all panoramic radiographs assessed under the same conditions?
17	Have panoramic radiographs been assessed from at least two assessors independently?
18	Have findings been reported on both patient level and panoramic radiograph image level, if applicable (multiple findings per image clarification)?
19	In case categorizations / poolings of findings have been made, have also separate individual findings been reported?
20	Have all possible findings that could be found in the particular panoramic radiograph been reported?
21	Has an appropriately large sample been analyzed?
22	Has the prevalence of observed findings been assessed?

Appendix 5. List of identified studies with their exclusion / inclusion status.

Nr	Paper	Status
1	Kandasamy S, Greene CS, Obrez A. An evidence-based evaluation of the concept of centric relation in the 21st century. QUINTESSENCE INTERNATIONAL. 2018;49(9):755-60.	Exclusion by title
2	King NM, Shaw L. VALUE OF BITEWING RADIOGRAPHS IN DETECTION OF OCCLUSAL CARIES. COMMUNITY DENTISTRY AND ORAL EPIDEMIOLOGY. 1979;7(4):218-21.	Exclusion by title
3	Akarslan ZZ, Akdevelioglu M, Güngör K, Erten H. A comparison of the diagnostic accuracy of bitewing, periapical, unfiltered and filtered digital panoramic images for approximal caries detection in posterior teeth. DENTOMAXILLOFACIAL RADIOLOGY. 2008;37(8):458-63.	Exclusion by abstract
4	Akkaya N, Kansu Ö, Kansu H, Çagirankaya LB, Arslan U. Comparing the accuracy of panoramic and intraoral radiography in the diagnosis of proximal caries. DENTOMAXILLOFACIAL RADIOLOGY. 2006;35(3):170-4.	Exclusion by abstract
5	Albert DGD, Gomes ACA, Vasconcelos BCE, Silva E, Holanda GZ. Comparison of orthopantomographs and conventional tomography images for assessing the relationship between impacted lower third molars and the mandibular canal. JOURNAL OF ORAL AND MAXILLOFACIAL SURGERY. 2006;64(7):1030-7.	Exclusion by abstract
6	Bahaziq A, Jadu FM, Jan AM, Baghdady M, Feteih RM. A Comparative Study of the Examination Pattern of Panoramic Radiographs Using Eye-tracking Software. J Contemp Dent Pract. 2019;20(12):1436-41.	Exclusion by abstract
7	Bille ML, Kvetny MJ, Kjaer I. A possible association between early apical resorption of primary teeth and ectodermal characteristics of the permanent dentition. Eur J Orthod. 2008;30(4):346-51.	Exclusion by abstract
8	Bille ML, Nolting D, Kvetny MJ, Kjaer I. Unexpected early apical resorption of primary molars and canines. Eur Arch Paediatr Dent. 2007;8(3):144-9.	Exclusion by abstract
9	Bissoon A, Bandoo C, Naraynsingh C, Mohamed S. Canalis Sinuosus Mimicking Periapical Pathology on, Radiographic Assessment. J Endod. 2024;50(8):1159-62.	Exclusion by abstract
10	Biun J, Lok V, Koong B, Huang TC. A significant incidental finding on cone beam computed tomography: multiple myeloma. Aust Dent J. 2019;64(3):293-6.	Exclusion by abstract
11	Carneiro NCR, Abreu LG, Milagres RMC, Amaral TMP, Flores-Mir C, Pordeus IA, et al. Dental and maxillomandibular incidental findings in panoramic radiography among individuals with mucopolysaccharidosis: a cross-sectional study. J Appl Oral Sci. 2021;29:e20200978.	Exclusion by abstract
12	Couto DAF, Tanaka JLO, Souza PHC, Ignacio SA, Carneiro E, Alanis LRA. Comparison of Panoramic Radiography and Cone Beam Computed Tomography for Lower Third Molar Assessment Among Different Dental Specialists. JOURNAL OF ORAL AND MAXILLOFACIAL SURGERY. 2025;83(8):1000-11.	Exclusion by abstract
13	Dayo A, Miles D, Boesze-Battaglia K, Sollecito T, Mupparapu M, Corby P. Trends Between Periodontitis and Medial Arterial Calcification in Undiagnosed Type II Diabetes Mellitus. Journal of Clinical and Translational Science. 2023;7:104.	Exclusion by abstract
14	Dias MJM, Franco A, Junqueira JLC, Fayad FT, Pereira PH, Oenning AC. Marginal bone loss in the second molar related to impacted mandibular third molars: comparison between panoramic images and cone beam computed tomography. MEDICINA ORAL PATOLOGIA ORAL Y CIRUGIA BUCAL. 2020;25(3):E395-E402.	Exclusion by abstract
15	Drew SJ, Cho JS. Fibula Free Flap Reconstruction of the Maxilla Leading to Extracapsular Ankylosis of the Mandible. Journal of Oral and Maxillofacial Surgery. 2022;80(4):767-74.	Exclusion by abstract
16	Espelid I, Tveit AB, Fjelltveit A. VARIATIONS AMONG DENTISTS IN RADIOGRAPHIC DETECTION OF OCCLUSAL CARIES. CARIES RESEARCH. 1994;28(3):169-75.	Exclusion by abstract
17	Espelid I, Tveit AB. A comparison of radiographic occlusal and approximal caries diagnoses made by 240 dentists. ACTA ODONTOLOGICA SCANDINAVICA. 2001;59(5):285-9.	Exclusion by abstract
18	Ferguson JW, Evans RIW, Cheng LHH. DIAGNOSTIC-ACCURACY AND OBSERVER PERFORMANCE IN THE DIAGNOSIS OF ABNORMALITIES IN THE ANTERIOR MAXILLA - A COMPARISON OF PANORAMIC WITH INTRAORAL RADIOGRAPHY. BRITISH DENTAL JOURNAL. 1992;173(8):265-71.	Exclusion by abstract
19	Katheria BC, Kau CH, Tate R, Chen JW, English J, Bouquot J. Effectiveness of Impacted and Supernumerary Tooth Diagnosis from Traditional Radiography Versus Cone Beam Computed Tomography. PEDIATRIC DENTISTRY. 2010;32(4):304-9.	Exclusion by abstract
20	Kumar M, Acharya S, Chhaparwal Y. Impaction of second premolar and primary molar in an inverse relation: A unique radiographic presentation. Dental and Medical Problems. 2016;53(4):570-2.	Exclusion by abstract
21	MacDonald-Jankowski DS, Li TK. Ossifying fibroma in a Hong Kong community: the clinical and radiological features and outcomes of treatment. Dentomaxillofac Radiol. 2009;38(8):514-23.	Exclusion by abstract
22	Makrygiannakis MA, Giannakopoulos K, Kavadella A, Paraskevis D, Kaklamanos EG. Diagnostic accuracy of an artificial intelligence-based software in detecting supernumerary and congenitally missing teeth in panoramic radiographs. EUROPEAN JOURNAL OF ORTHODONTICS. 2025;47(4).	Exclusion by abstract
23	Makrygiannakis MA, Kaklamanos EG. Assessment of AI software's diagnostic accuracy in identifying impacted teeth in panoramic radiographs. EUROPEAN JOURNAL OF ORTHODONTICS. 2025;47(5).	Exclusion by abstract
24	Manor Y, Abir R, Manor A, Kaffe I. Are different imaging methods affecting the treatment decision of extractions of mandibular third molars? DENTOMAXILLOFACIAL RADIOLOGY. 2016;46(1).	Exclusion by abstract
25	Matzen LH, Christensen J, Hintze H, Schou S, Wenzel A. Diagnostic accuracy of panoramic radiography, stereo-scanography and cone beam CT for assessment of mandibular third molars before surgery. ACTA ODONTOLOGICA SCANDINAVICA. 2013;71(6):1391-8.	Exclusion by abstract
26	Matzen LH, Petersen LB, Wenzel A. Radiographic methods used before removal of mandibular third molars among randomly selected general dental clinics. DENTOMAXILLOFACIAL RADIOLOGY. 2016;45(4).	Exclusion by abstract
27	Matzen LH, Schou S, Christensen J, Hintze H, Wenzel A. Audit of a 5-year radiographic protocol for assessment of mandibular third molars before surgical intervention. DENTOMAXILLOFACIAL RADIOLOGY. 2014;43(8).	Exclusion by abstract
28	Mitchell OR, Singh RP. Conservative technique for enucleation of a large dentigerous cyst through bony fenestrations. British Journal of Oral and Maxillofacial Surgery. 2019;57(7):704-5.	Exclusion by abstract
29	Monaco G, Montevecchi M, Bonetti GA, Gatto MRA, Checchi L. Reliability of panoramic radiography in evaluating the topographic relationship between the mandibular canal and impacted third molars. JOURNAL OF THE AMERICAN DENTAL ASSOCIATION. 2004;135(3):312-8.	Exclusion by abstract
30	Nakagawa Y, Ishii H, Nomura Y, Watanabe NY, Hoshiba D, Kobayashi K, et al. Third molar position: Reliability of panoramic radiography. JOURNAL OF ORAL AND MAXILLOFACIAL SURGERY. 2007;65(7):1303-8.	Exclusion by abstract
31	Nakamoto T, Taguchi A, Ohtsuka M, Suei Y, Fujita M, Tanimoto K, et al. Dental panoramic radiograph as a tool to detect postmenopausal women with low bone mineral density: untrained general dental practitioners' diagnostic performance. Osteoporos Int. 2003;14(8):659-64.	Exclusion by abstract
32	Rangel FA, Maal TJJ, de Koning MJJ, Bronkhorst EM, Bergé SJ, Kuijpers-Jagtman AM. Integration of digital dental casts in cone beam computed tomography scans-a clinical validation study. CLINICAL ORAL INVESTIGATIONS. 2018;22(3):1215-22.	Exclusion by abstract
33	Resnick CM, Dentino KM, Garza R, Padwa BL. A Management Strategy for Idiopathic Bone Cavities of the Jaws. J Oral Maxillofac Surg. 2016;74(6):1153-8.	Exclusion by abstract
34	Ruy Carneiro NCR, Abreu LG, Milagres RMC, Amaral TMP, Flores-Mir C, Pordeus I, et al. Dental and maxillomandibular incidental findings in panoramic radiography among individuals with mucopolysaccharidosis: A crosssectional study. Journal of Applied Oral Science. 2021;29.	Exclusion by abstract
35	Sewell J, Drage N, Brown J. The use of panoramic radiography in a dental accident and emergency department. Dentomaxillofacial Radiology. 2001;30(5):260-3.	Exclusion by abstract
36	Simiyu BN, Butt F, Dimba EA, Wagaiyu EG, Awange DO, Guthua SW, et al. Keratocystic odontogenic tumours of the jaws and associated pathologies: a 10-year clinicopathologic audit in a referral teaching hospital in Kenya. J Craniomaxillofac Surg. 2013;41(3):230-4.	Exclusion by abstract
37	Taylor NG, Jones AG. ARE ANTERIOR OCCLUSAL RADIOGRAPHS INDICATED TO SUPPLEMENT PANORAMIC RADIOGRAPHY DURING AN ORTHODONTIC ASSESSMENT. BRITISH DENTAL JOURNAL. 1995;179(10):377-81.	Exclusion by abstract
38	Tkaczuk AT, Bhatti M, Caccamese JF, Jr., Ord RA, Pereira KD. Cystic Lesions of the Jaw in Children: A 15-Year Experience. JAMA Otolaryngol Head Neck Surg. 2015;141(9):834-9.	Exclusion by abstract
39	Vinayahalingam S, van Nistelrooij N, Xi T, Heiland M, Bressem K, Rendenbach C, et al. Detection of carotid plaques on panoramic radiographs using deep learning. J Dent. 2024;151:105432.	Exclusion by abstract
40	Wenzel A, Aagaard E, Sindet-Pedersen S. Evaluation of a new radiographic technique: diagnostic accuracy for mandibular third molars. DENTOMAXILLOFACIAL RADIOLOGY. 1998;27(5):255-63.	Exclusion by abstract
41	Wenzel A, Fejerskov O. VALIDITY OF DIAGNOSIS OF QUESTIONABLE CARIES LESIONS IN OCCLUSAL SURFACES OF EXTRACTED 3RD MOLARS. CARIES RESEARCH. 1992;26(3):188-94.	Exclusion by abstract
42	Winocur E, Reiter S, Krichmer M, Kaffe I. Classifying degenerative joint disease by the RDC/TMD and by panoramic imaging: a retrospective analysis. J Oral Rehabil. 2010;37(3):171-7.	Exclusion by abstract
43	Witcher TP, Brand S, Gwilliam JR, McDonald F. Assessment of the anterior maxilla in orthodontic patients using upper anterior occlusal radiographs and dental panoramic tomography: a comparison. ORAL SURGERY ORAL MEDICINE ORAL PATHOLOGY ORAL RADIOLOGY AND ENDODONTOLOGY. 2010;109(5):765-74.	Exclusion by abstract
44	Abdalla-Aslan R, Friedlander-Barenboim S, Aframian DJ, Maly A, Nadler C. Ameloblastoma incidentally detected in cone-beam computed tomography sialography: A case report and review of the literature. J Am Dent Assoc. 2018;149(12):1073-80.	Excluded; case report / series
45	Açikgöz A. Bilateral bifid mandibular condyle: a case report. J Oral Rehabil. 2006;33(10):784-7.	Excluded; case report / series
46	Agarwal S, Anand A, Ranjan P, Meena VP, Ray A, Dutta R, et al. Case of mucormycosis of mandible after self-extraction of teeth incidentally detected to have chronic granulomatous disease: Case report and literature review. Medical Mycology Case Reports. 2020;28:55-9.	Excluded; case report / series
47	Agrawal A, Singh A, Bhake A, Vagha S. Plasma cell gingivitis: A rare perpetuating entity. Indian Journal of Forensic Medicine and Toxicology. 2020;14(4):6729-31.	Excluded; case report / series
48	Ahn SY, Kim NH, Kim S, Karabucak B, Kim E. Computer-aided Design/Computer-aided Manufacturing-guided Endodontic Surgery: Guided Osteotomy and Apex Localization in a Mandibular Molar with a Thick Buccal Bone Plate. JOURNAL OF ENDODONTICS. 2018;44(4):665-70.	Excluded; case report / series
49	Ai CJ, Jabar NA, Lan TH, Ramli R. Case series: Mandibular canal enlargement: Clinical & radiological characteristics. Journal of Clinical Imaging Science. 2017;7(1):1-7.	Excluded; case report / series
50	Ajila V, Gopakumar R, Hegde S, Babu S, Harini K. Unusual presentation of solitary bone cyst - A case report. Nitte University Journal of Health Science. 2014;4(1):135-7.	Excluded; case report / series
51	Akbarian Tefaghi F, Adams A, Makdissi J. Utilising previous radiographs: the principle of dose optimisation. BJR Case Rep. 2020;6(4):20200018-.	Excluded; case report / series
52	Alessandri Bonetti G, Incerti Parenti S, Daprile G, Montevecchi M. Failure after closed traction of an unerupted maxillary permanent canine: Diagnosis and treatment planning. Am J Orthod Dentofacial Orthop. 2011;140(1):121-5.	Excluded; case report / series
53	Al-Haj Husain A, Salzer E, Stadlinger B. Challenges in the diagnosis and management of soft tissue tumors of the oral cavity. Swiss Dent J. 2024;134(4):27-36.	Excluded; case report / series
54	Allen GA, Liston SL. Rhinolith: unusual appearance on panoramic radiograph. J Oral Surg. 1979;37(1):54-5.	Excluded; case report / series
55	Almog DM, Illig KA, Khin M, Green RM. Unrecognized carotid artery stenosis discovered by calcifications on a panoramic radiograph. J Am Dent Assoc. 2000;131(11):1593-7.	Excluded; case report / series
56	Alpaslan S, Ozbek M, Hersek N, Kanli A, Avcu N, Firat M. Bilateral bifid mandibular condyle. Dentomaxillofac Radiol. 2004;33(4):274-7.	Excluded; case report / series
57	Al-Rabai NAA, Assiri KI, Al Almai MI, Sandeepa NC, Ajmal M, Divakar DD. Mucoepidermoid carcinoma of minor salivary gland in a patient with asymptomatic palatal swelling-A case report. Journal of Pharmacy and Bioallied Sciences. 2022;14(5):S1046-S9.	Excluded; case report / series
58	Amin D, Magliocca KR, Abramowicz S. Calcifying odontogenic cyst: Report of 2 cases and review of literature. Oral and Maxillofacial Surgery Cases. 2020;6(2).	Excluded; case report / series
59	Amin Z, Colosi D, Odingo N, Reddy R. A rare case of unilateral double Stafne bone defects and literature review. Oral Radiology. 2024;40(3):454-61.	Excluded; case report / series
60	Anbiaee N, Tafakhori Z. Early diagnosis of progressive systemic sclerosis (scleroderma) from a panoramic view: report of three cases. Dentomaxillofac Radiol. 2011;40(7):457-62.	Excluded; case report / series

61	Anchlia S, Bahl S, Vyas S, Raju GS. Unicystic plexiform ameloblastoma with mural proliferation: a full-blown lesion. BMJ Case Rep. 2016;2016.	Excluded; case report / series
62	Aoki EM, Abdala-Júnior R, Nagano CP, Mendes EB, Oliveira JX, Lourenço SV, et al. Simple Bone Cyst Mimicking Stafne Bone Defect. J Craniofac Surg. 2018;29(6):e570-e1.	Excluded; case report / series
63	Apipi M, Shanmuhasuntharam P. Calcifying cystic odontogenic tumour with odontoma formation: A case report and literature review. International Journal of Oral and Maxillofacial Surgery. 2017;46:299.	Excluded; case report / series
64	Arellano-Villalón M, Navarro P, Arias A, Fuentes R. Hallazgo radiográfico de cuerpo extraño en piso de seno maxilar de sujeto asintomático. Int j odontostomatol (Print). 2020;14(2):150-3.	Excluded; case report / series
65	Arishiya Thapasum F, Mohammed F. Susuk- Black magic exposed "White" by dental radiographs. Journal of Clinical and Diagnostic Research. 2014;8(7):3-4.	Excluded; case report / series
66	Baroni C, Farneti M, Stea S, Rimondini L. AMELOBLASTIC FIBROMA AND IMPACTED MANDIBULAR 1ST MOLAR - A CASE-REPORT. ORAL SURGERY ORAL MEDICINE ORAL PATHOLOGY ORAL RADIOLOGY AND ENDODONTICS. 1992;73(5):548-9.	Excluded; case report / series
67	Bassetti R, Tomasetti P, Crameri M, Kuttenberger J. [Benign odontogenic tumor in the lower jaw: A case report]. Swiss Dent J. 2016;126(4):361-70.	Excluded; case report / series
68	Bayram B, Deniz K, Özsoy O, Uçkan S. A simple surgical approach for management of the rhinoliths: Case report. Journal of Oral and Maxillofacial Surgery. 2011;69(5):1403-7.	Excluded; case report / series
69	Belardo E, Velasco I, Guerra A, Rosa E. Mandibular ameloblastoma in a 10-year-old child: case report and review of the literature. Int j odontostomatol (Print). 2012;6(3):331-6.	Excluded; case report / series
70	Borumandi F, Lukas H, Yousefi B, Gaggl A. Maxillary sinus osteoma: From incidental finding to surgical management. J Oral Maxillofac Pathol. 2013;17(2):318-.	Excluded; case report / series
71	Bouhoute M, Taleb B. Cystic degeneration in cemento-ossifying fibroma: Diagnosis challenge and conservative management - Case report. International Journal of Surgery Case Reports. 2022;90.	Excluded; case report / series
72	Breen GH, Addante R. A recurrent traumatic bone cyst: report of case. ASDC J Dent Child. 1994;61(2):141-5.	Excluded; case report / series
73	Breen GH, Addante R. A RECURRENT TRAUMATIC BONE-CYST - REPORT OF CASE. JOURNAL OF DENTISTRY FOR CHILDREN. 1994;61(2):141-5.	Excluded; case report / series
74	Brooks JK, Alshami H, Younis RH, Jang H, Yousef N, Altajjar H, et al. Diminutive compound odontoma in a child: a case report with emphasis on early detection. Gen Dent. 2024;72(1):43-5.	Excluded; case report / series
75	Brooks JK, Gardullo PM, Parsa A, Price JB. Incidental detection of bilateral cochlear implants on panoramic radiography: Case report associated with antecedent traumatic brain injury. Special Care in Dentistry. 2024;44(3):719-21.	Excluded; case report / series
76	Brooks JK, Kumar V, Parsa A, Price JB. Pseudopathologic findings on panoramic radiographs and utility of follow-up cone beam computed tomographic scans: a case series. Gen Dent. 2023;71(5):34-7.	Excluded; case report / series
77	Brooks JK, Macauley MR, Price JB. Concurrent giant sialoliths within the submandibular gland parenchyma and distal segment of Wharton's duct: Novel case report. Gerodontology. 2021;38(4):437-40.	Excluded; case report / series
78	Bugshan AS, Alsaati MA, Syed FA, Almulhim KS, Abdulhady AI. Incidental Diagnosis on Orthopantomography of Langerhans Cell Histiocytosis with Multifocal Jaw Involvement: A Case Report of Single-System Disease. Am J Case Rep. 2020;21:e928307.	Excluded; case report / series
79	Byung-Do LEE. A case report of incidental finding of fungus ball on CBCT of maxillary sinus in treatment planning of dental implant. Korean Journal of Oral and Maxillofacial Radiology. 2010:93-7.	Excluded; case report / series
80	Canger EM, Kayipmaz S, Celenk P. Bilateral dens invaginatus in the mandibular premolar region. Indian J Dent Res. 2009;20(2):238-40.	Excluded; case report / series
81	Cardona Mejía SE, Uribe Escobar A, García García CA. Tonsilolito gigante: hallazgo inesperado. Reporte de caso. Acta otorrinolaringol cir cuello (En línea). 2023;51(3):228-31.	Excluded; case report / series
82	Chaparro González NT, Alamancos Torres IC, Hermoso Solórzano L, Rodríguez Azuaje RP, Jiménez Zerpa AC. Herencia autosómica recesiva en paciente no sindrómico con múltiples dientes supernumerarios. Acta odontol venez. 2014;52(1).	Excluded; case report / series
83	Chawla R, Garcha P, Lyne A, Sheriteh Z. Evidence that ectopic upper third molars can result in upper second molar impactions: A case series. Journal of Orthodontics. 2023.	Excluded; case report / series
84	Cochrane SM, Clark JR, Hunt NP. Late developing supernumerary teeth in the mandible. Br J Orthod. 1997;24(4):293-6.	Excluded; case report / series
85	Consolo U, Tognacci S, Barausse C, Salgarelli A, Bellini P. Enucleation of a multilocular odontogenic keratocyst using sagittal osteotomy: A case report. Oral and Maxillofacial Surgery Cases. 2020;6(2).	Excluded; case report / series
86	Corchero-Martín G, Gonzalez-Terán T, García-Reija MF, Sánchez-Santolino S, Saiz-Bustillo R. Bifid condyle: case report. Med Oral Patol Oral Cir Bucal. 2005;10(3):277-9.	Excluded; case report / series
87	Cossellu G, Angiero F, Farronato G. Complete pre-eruptive idiopathic crown resorption. Pediatr Dent. 2014;36(7):147-50.	Excluded; case report / series
88	Counihan KP, O'Connell AC. Case report: pre-eruptive intra-coronal radiolucencies revisited. Eur Arch Paediatr Dent. 2012;13(4):221-6.	Excluded; case report / series
89	Cowan DF, Ferguson MM. Bifid mandibular condyle. Dentomaxillofacial Radiology. 1997;26(1):70-3.	Excluded; case report / series
90	Cutmore N, Chengot P. A case of sclerosing odontogenic carcinoma: a rare entity. Diagnostic Histopathology. 2023;29(4):232-5.	Excluded; case report / series
91	da Costa ED, Roque-Torres GD, Peyneau PD, Godolfim LR, Haiter Neto F, Almeida SM. Simple bone cyst: rare incidental finding in the mandibular condyle by cone beam computed tomography. Gen Dent. 2018;66(1):54-6.	Excluded; case report / series
92	de Castro J-F-L, Correia A-V-L, Santos L-A-M, Guerra L-A-P, Ramos-Perez F-M-d-M, Perez D-E-dC. Ameloblastic fibroma: A rare case appearing as a mixed radiographic image. J Clin Exp Dent. 2014;6(5):e583-7.	Excluded; case report / series
93	Delantoni A, Antoniades I. The iatrogenic fracture of the coronoid process of the mandible. A review of the literature and case presentation. Cranio - Journal of Craniomandibular Practice. 2010;28(3):200-4.	Excluded; case report / series
94	Delantoni A, Lyroudia K, Rafailidis V, Chryssogonidis I. Pseudohypoparathyroidism diagnosed in adulthood: maxillofacial clinical and radiographic findings. Oral Radiology. 2017;33(2):153-6.	Excluded; case report / series
95	Delantoni A, Papademitriou P. An unusually large asymptomatic periapical lesion that presented as a random finding on a panoramic radiograph. Oral Surg Oral Med Oral Pathol Oral Radiol Endod. 2007;104(2):e62-5.	Excluded; case report / series
96	Deshpande P, Patil K, Solayappan E, Doggalli N. Unveiling the enigma: bilateral Stafne bone defect as an incidental discovery. European Journal of Anatomy. 2025;29(1):121-5.	Excluded; case report / series
97	Devang Divakar D, Mavinapalla S, Al Kheraif AA, Ramakrishnaiah R, Abd Rahim MF, Khan AA. Incidental radiographic finding of the concealed art of susuk. Med Sci Law. 2016;56(3):230-2.	Excluded; case report / series
98	Di Donna E, Keller LM, Neri A, Perez A, Lombardi T. Maxillary Distomolar Associated with Dentigerous Cyst: An Unusual Entity. ORAL. 2022;2(1):1-6.	Excluded; case report / series
99	Ducommun F, Bornstein MM, Bosshardt D, Katsaros C, Dula K. Diagnosis of tooth ankylosis using panoramic views, cone beam computed tomography, and histological data: a retrospective observational case series study. EUROPEAN JOURNAL OF ORTHODONTICS. 2018;40(3):231-8.	Excluded; case report / series
100	Dvori S, Shohat Y, Taicher S. [Simple bone cyst in the mandible--a rare occurrence in an elderly patient]. Refuat Hapeh Vehashinayim (1993). 2006;23(1):27-30, 69.	Excluded; case report / series
101	Elseyoufi M, Coleman H, Tsakiris P. Chondromyxoid fibroma of the mandible: A case report. Advances in Oral and Maxillofacial Surgery. 2021;1.	Excluded; case report / series
102	Ertas ET, Mavili E, Sisman Y, Sahman H, Etoz M, Sekerci AE. Incidental findings of carotid artery stenosis detected by calcifications on panoramic radiographs: Report of three cases. Oral Radiology. 2010;26(2):116-21.	Excluded; case report / series
103	Faria VdA, Vilela FP, Wendich GC, Watanabe MGdC, Pacheco JA, Paranhos HdFO, et al. Idiopathic exostosis: rarity on maxillary sinus. Rev Cient CRO-RJ (Online). 2019;4(1):102-6.	Excluded; case report / series
104	Fitzgerald J, Ziegler ME, Green PT, Neville BW. Calcified facial and maxillary arteries: Incidental radiographic findings indicative of Mönckeberg arteriosclerosis. J Am Dent Assoc. 2021;152(11):943-6.	Excluded; case report / series
105	Florin W, Mandel L. Stafne's bone cavity anterior mandible. Case report. N Y State Dent J. 2010;76(3):36-8.	Excluded; case report / series
106	Freire JdOA, Bianco GdS, Imada TSN, Salzedas LMP, Ikuta CRS. Radiographic examination in the diagnosis of a curious case associating odontoma to agenesis and supernumerary teeth. RGO (Porto Alegre). 2021;69:e20210048-e.	Excluded; case report / series
107	Freitas F, Rocha F, Moreira A, Martins J, Caramês J, Amaral Mednes R. Diagnosis and management of a large mandibular cystic lesion. Oral Diseases. 2014;20:32.	Excluded; case report / series
108	Friedrich RE, Grob TJ, Hollants S, Zustin J, Spaepen M, Mautner VF, et al. Recurrent multilocular mandibular giant cell granuloma in neurofibromatosis type 1: Evidence for second hit mutation of NF1 gene in the jaw lesion and treatment with curettage and bone substitute materials. Journal of Cranio-Maxillofacial Surgery. 2016;44(8):1054-60.	Excluded; case report / series
109	Furuuchi T, Kochi S, Sasano T, Iikubo M, Komai S, Igari K. Morphologic characteristics of masseter muscle in cleidocranial dysplasia: a report of 3 cases. Oral Surg Oral Med Oral Pathol Oral Radiol Endod. 2005;99(2):185-90.	Excluded; case report / series
110	Gadhia K, Bunyan R, Chan CH. Multiple radio-opacities in an OPG: a case report of cavernous haemangioma of temporalis muscle with multiple phleboliths. Dent Update. 2011;38(10):711-3.	Excluded; case report / series
111	Garg AK, Chaudhary A, Tewari RK, Bariar LM, Agrawal N. Coincidental diagnosis of tuberculous lymphadenitis: a case report. Aust Dent J. 2014;59(2):258-63.	Excluded; case report / series
112	Georgiou AF, Yeoh SC, Coleman H. Hidden mandibular pathology in a patient planned for radiotherapy highlighting the need for dental screening. Journal of Radiotherapy in Practice. 2011;10(1):65-8.	Excluded; case report / series
113	Ghousia S, Firdoose N. CBCT Evaluation of Unilateral Accessory Mental Foramina: under Reported Anatomic Variant in Children. Int j odontostomatol (Print). 2022;16(3):441-6.	Excluded; case report / series
114	Girgis S, Cheng L, Gillet D. A rhinolith mimicking a toothache. International Journal of Oral and Maxillofacial Surgery. 2015;44:e195-e6.	Excluded; case report / series
115	Girgis S, Cheng L, Gillett D. Rhinolith mimicking a toothache. International Journal of Surgery Case Reports. 2015;14:66-8.	Excluded; case report / series
116	Gonzalez SM, Spalding PM, Payne JB, Giannini PJ. A dentigerous cyst associated with bilaterally impacted mandibular canines in a girl: A case report. Journal of Medical Case Reports. 2011;5.	Excluded; case report / series
117	Grocholewicz K, Janiszewska-Olszowska J, Kacperski Ł. Odontogenous sinus tract to the chin--case report. Ann Acad Med Stetin. 2013;59(2):86-9.	Excluded; case report / series

118	Gulsahi A, Gulsahi K, Ungor M. Invasive cervical resorption: clinical and radiological diagnosis and treatment of 3 cases. Oral Surg Oral Med Oral Pathol Oral Radiol Endod. 2007;103(3):e65-72.	Excluded; case report / series
119	Günaçar DN, Köse TE, Atasoy Sındıraç A, Uyanık SA. Can Myelography Residues Have an Incidental Finding on Panoramic Radiography? Two Case Reports. J Oral Maxillofac Surg. 2021;79(9):1891.e1-.e4.	Excluded; case report / series
120	Gunduz K, Avsever H, Karacayli U. Bilateral bifid condylar process. Int j morphol. 2010;28(3):941-4.	Excluded; case report / series
121	Gündüz K, Çelenk P, Alkan A. Intranasal radiopacities: Incidental panoramic radiograph findings. Oral Radiology. 2010;26(1):41-5.	Excluded; case report / series
122	Hall AM, Orth D. The solitary bone cyst. Report of two cases. Oral Surg Oral Med Oral Pathol. 1976;42(2):164-8.	Excluded; case report / series
123	Hamamoto M, Shimamoto H, Oya K, Fukuda Y, Kreïborg S, Mallya SM, et al. MR appearance of a rare ameloblastic fibroma with formation of dental hard tissues with histopathologic correlation: a case report. Oral Radiology. 2023;39(1):220-4.	Excluded; case report / series
124	Hameed O, Gwilliam J, Whaites E. Odontogenic keratocyst: An incidental finding during an orthodontic examination. J Orthod. 2020;47(3):245-50.	Excluded; case report / series
125	Hania M, Sharif MO. Maxillary sinus osteoma: A case report and literature review. J Orthod. 2020;47(3):240-4.	Excluded; case report / series
126	HarshaVardhan BG, Vignesswary A, Kumar VJ, SaraswathiGopal K. Lateral periodontal cyst-An unusual radiographic presentation. Biomedicine (India). 2015;35(1):103-6.	Excluded; case report / series
127	Hasnaini M, Ng SY. Extensive temporal bone pneumatization: incidental finding in a patient with TMJ dysfunction. Dent Update. 2000;27(4):187-9.	Excluded; case report / series
128	Hatakeyama D, Tamaoki N, Iida K, Yonemoto K, Kato K, Makita H, et al. Simple bone cyst of the mandibular condyle in a child: report of a case. J Oral Maxillofac Surg. 2012;70(9):2118-23.	Excluded; case report / series
129	Henriques JC, Kreich EM, Rosa RR, Castilho JC, de Moraes LC, de Moraes ME. Noninvasive aspergillosis as a maxillary antrolith: report of a rare case. Quintessence Int. 2012;43(2):143-6.	Excluded; case report / series
130	High AS. Covert foreign body simulating neoplasm. Dentomaxillofacial Radiology. 1989;18(3):136-7.	Excluded; case report / series
131	Horne RP, Meara DJ, Granite EL. Idiopathic bone cavities of the mandible: an update on recurrence rates and case report. Oral Surg Oral Med Oral Pathol Oral Radiol. 2014;117(2):e71-3.	Excluded; case report / series
132	Hutton SB, Kalamchi S, Wright JM. An incidentally discovered radiolucency in the posterior mandible. Oral Surg Oral Med Oral Pathol Oral Radiol. 2012;113(1):17-20.	Excluded; case report / series
133	Igoumenakis D, Athanasiou S, Mourouzis C, Machaira E, Mezitis M. An incidentally discovered radiolucency in the posterior maxilla. Oral Surg Oral Med Oral Pathol Oral Radiol. 2014;118(5):513-8.	Excluded; case report / series
134	Indermun S, Simpson J, Pedro-Beech K, Alwan J, van Rensburg LJ. Radiologic and Pathologic Insights into Medication-Related Osteonecrosis of the Jaw in Myeloma Patients: A Report of 3 Cases. Radiology Case Reports. 2025;20(10):5107-15.	Excluded; case report / series
135	Indini A, Luksch R, Patriarca C, Petracco G, Ronchi P, Colombo L, et al. Incidental ameloblastoma diagnosed after treatment for childhood tumor. Journal of Pediatric Surgery Case Reports. 2017;23:50-2.	Excluded; case report / series
136	Israr AR, Zamir A, Israr M. Proliferative Myositis in Mandible: A Case Report. Journal of the College of Physicians and Surgeons Pakistan. 2025;35(1):114-6.	Excluded; case report / series
137	Jain RK, Frommer HH. Incidental finding of antroliths in panoramic radiography. N Y State Dent J. 1982;48(8):530-1.	Excluded; case report / series
138	Joshi A, Goel M, Fating N, Dawane P. Multiple unerupted impacted supernumerary teeth: a rare case report of father and son. Int j med surg sci (Print). 2016;3(2):863-7.	Excluded; case report / series
139	Kalra A, Sheehy EC, Johnson J, McDonald F. A Bag of Marbles: A Fascinating Compound Odontoma of the Maxilla. Pediatr Dent. 2018;40(2):140-2.	Excluded; case report / series
140	Kamatani T, Tatemoto Y, Tateishi Y, Yamamoto T. Isolated metastasis from hepatocellular carcinoma to the mandibular condyle with no evidence of any other metastases: a case report. Br J Oral Maxillofac Surg. 2008;46(6):499-501.	Excluded; case report / series
141	Kannan S, Muthusamy S, Muthu K, Sidhu P. Multiple bony overgrowths in the mouth - Report of two cases. Clinical Cases in Mineral and Bone Metabolism. 2015;12(3):260-1.	Excluded; case report / series
142	Kanungo M, Khandelwal V, Nayak UA, Nayak PA. Multiple idiopathic apical root resorption. BMJ Case Rep. 2013;2013.	Excluded; case report / series
143	Khatib B, Pogrel MA. Desmoplastic fibroma of the mandible in young children—a case series. International Journal of Oral and Maxillofacial Surgery. 2017;46(2):173-80.	Excluded; case report / series
144	Khonsari RH, Corre P, Bouguila J, Piot B. [Long-term follow-up of cemento-osseous dysplasia: a contralateral recurrence before radiotherapy]. Rev Stomatol Chir Maxillofac. 2011;112(1):51-3.	Excluded; case report / series
145	Kini Y, Kamat A, Navalkha K, Nayan S, Mandlik G, Gupta D. Co-existence of oral intra-muscular lipoma and lobular capillary hemangioma: A rare case report. International Journal of Surgery Case Reports. 2020;77:704-7.	Excluded; case report / series
146	Ko DE, Yoon JH, Kim AY, Song HM, Lee SB, Park WJ, et al. Cemento-osseous Dysplasia Mimicking Periapical Cyst After the Orthodontic Treatment of the Mandible. J Craniofac Surg. 2025;36(5):e516-e8.	Excluded; case report / series
147	Kuralt H, Fidler A, Blatnik A, Novaković S, Plavc G. Bilateral parotid glands aplasia: a case report and literature review. Oral Radiology. 2022;38(3):423-9.	Excluded; case report / series
148	Lee KC, Yoon AJ, Philipone EM, Peters SM. Stafne Bone Defect Involving the Ascending Ramus. J Craniofac Surg. 2019;30(4):e301-e3.	Excluded; case report / series
149	Loh FC, Yeo JF. Bifid mandibular condyle. Oral Surg Oral Med Oral Pathol. 1990;69(1):24-7.	Excluded; case report / series
150	Lovel SF, Lovelock DJ, Shaw AJ. An unusual intranasal foreign body in an unsuspecting 9-year old. International Journal of Paediatric Dentistry. 2005;15(1):78-81.	Excluded; case report / series
151	Luitel A, Rimal J, Maharjan IK, Niroula D, Regmee P. Even a Tooth Slips Sometimes. Kathmandu Univ Med J (KUMJ). 2016;14(55):285-7.	Excluded; case report / series
152	M B, Ramani P, Tks B, Warrier S A, Jeyakumaran S. Hemangioma of Tongue With Multiple Phleboliths Involving Multiple Spaces of the Right Side of the Head and Neck Region: A Case Report Along With the Importance of Specialized Imaging. Cureus. 2023;15(8):e44330-e.	Excluded; case report / series
153	MacDonald D, Li T, Leung SF, Curtin J, Yeung A, Martin MA. Extranodal lymphoma arising within the maxillary alveolus: a case report. Oral Surg Oral Med Oral Pathol Oral Radiol. 2017;124(3):e233-e8.	Excluded; case report / series
154	Maciel AP, Lopes IA, Tucunduva RMA, Simpione G, Da Silva Santos PS, Damante JH, et al. Contribution of the CBCT in the diagnosis and treatment plan of odontogenic maxillary sinusitis: Cases Reports. Revista Estomatologica Herediana. 2020;30(1):47-52.	Excluded; case report / series
155	Maheshwari N, Etikaala B, Syed AZ. Rhinolith: An incidental radiographic finding. IMAGING SCIENCE IN DENTISTRY. 2021;51(3):333-6.	Excluded; case report / series
156	Mainassara Chekaraou S, Rabe Amani AH, Habibou AM, Mascou M, Ali A, Djafarou AB, et al. Odontogenic cysts decompression: Myth or reality? A case report. Advances in Oral and Maxillofacial Surgery. 2024;15.	Excluded; case report / series
157	Makdissi J, Barez R, Whaites E, Brown J. Case report--Scanora imaging of an unusual unerupted tooth located entirely below the inferior dental canal. J Ir Dent Assoc. 2003;49(2):43-6.	Excluded; case report / series
158	Mamatha NS, Kedarnath NS, Singh M, Patel G. Accessory mental nerve: A case report. Journal of Clinical and Diagnostic Research. 2013;7(9):2078-9.	Excluded; case report / series
159	Marizan Nor MM, Yushar A, Razali M, Rahman RA, Ramli R. Incidental radiological findings of susuk in the orofacial region. Dentomaxillofacial Radiology. 2006;35(6):473-4.	Excluded; case report / series
160	Maspero C, Abate A, Inchingolo F, Dolci C, Cagetti MG, Tartaglia GM. Incidental Finding in Pre-Orthodontic Treatment Radiographs of an Aural Foreign Body: A Case Report. Children. 2022;9(3).	Excluded; case report / series
161	McIntyre G. Kissing molars: an unexpected finding. Dent Update. 1997;24(9):373-4.	Excluded; case report / series
162	McKinney SL, Lukes SM. Dentigerous cyst in a young child: A case report. Canadian Journal of Dental Hygiene. 2021;55(3):177-81.	Excluded; case report / series
163	McNamara CM, Garvey MT, Winter GB, McNamara T. Root abnormalities, talon cusps, dens invaginati with reduced alveolar bone levels: Case report. International Journal of Paediatric Dentistry. 1998;8(1):41-5.	Excluded; case report / series
164	Melloni F, Cadore L, Faccioni P, Balliu F, Costantini M, Beccherle M, et al. Precise Extraction of Impacted Supernumerary Teeth in the Mandibular Anterior Region: A Case Report. Journal of Applied Cosmetology. 2025;43(2):196-202.	Excluded; case report / series
165	Mizbah K, Gerlach N, Maal TJ, Bergé SJ, Meijer GJ. [Bifid and trifid mandibular canal. A coincidental finding]. Ned Tijdschr Tandheelkd. 2010;117(12):616-8.	Excluded; case report / series
166	Mohad N, Dabir A, Vahanwala J, Padhye M, Patwardhan J. Tuberculous osteomyelitis in condyle of mandible: A case report. Advances in Oral and Maxillofacial Surgery. 2021;2.	Excluded; case report / series
167	More CB, Das S, Gupta S, Patel P, Saha N. Stafne's bone cavity: A diagnostic challenge. Journal of Clinical and Diagnostic Research. 2015;9(11):ZD16-ZD9.	Excluded; case report / series
168	Mosca RC, Cabral MVG, Mantesso A. Bilateral tonsilloliths in a 77-year-old white man with edentulous jaws: A case report. Oral Radiology. 2006;22(1):34-6.	Excluded; case report / series
169	Moussa NT, Doscher J, Lane C, Woo L, Dhillon P, Rosenfeld J. Case Report: Incidental Finding of an Osteoblastoma of the Mandible. Journal of Oral and Maxillofacial Surgery. 2021;79(10):e92-e3.	Excluded; case report / series
170	Mukdad M, Barut O, Sjöström M. Intralesional corticosteroid injections as first option for management of giant cell lesion of the lower jaw in a 56-year-old patient: A case report and brief literature review. Oral and Maxillofacial Surgery Cases. 2022;8(4).	Excluded; case report / series
171	Mulimani P, Kumbargere Nagraj S, Koshy C. Application of CBCT in orthodontics: Hyper-pneumatized maxillary sinus associated with impacted mesiodens - Case report. International Journal of Clinical Dentistry. 2017;10(4):271-84.	Excluded; case report / series
172	Münevveroglu AP, Aydin KC. Stafne bone defect: report of two cases. Case Rep Dent. 2012;2012:654839-.	Excluded; case report / series
173	Mupparapu M, Binder RE, Duarte F. Hereditary cranium bifidum persisting as enlarged parietal foramina (Catlin marks) on cephalometric radiographs. Am J Orthod Dentofacial Orthop. 2006;129(6):825-8.	Excluded; case report / series
174	Myers DT, Karvelis KC. Incidental finding of periodontal disease on bone scan. Clin Nucl Med. 1994;19(7):644-5.	Excluded; case report / series

175	Narayananan VS, Ashok L, Mamatha GP, Rajeshwari A, Prasad SS. Pyle's disease: an incidental finding in a routine dental patient. Dentomaxillofac Radiol. 2006;35(1):50-4.	Excluded; case report / series
176	Natarajan S, Madhankumar S, Jeyapalan K, Athiban I, Elengkumaran S, Periyakaruppiah KPS. Disto-angular transmigrated impacted mandibular molar with enostosis: A rare intraoral lesion. Journal of Pharmacy and Bioallied Sciences. 2015;7(6):S791-S3.	Excluded; case report / series
177	Neha S, Santosh M, Sachin MG, Poonam SR, Simranjit S, Abdul KA. Adenomatoid odontogenic tumour: An enigma. Saudi Dental Journal. 2018;30(1):94-6.	Excluded; case report / series
178	Ommerborn MA, John G, Becker J, Preetz J, Gabris S. Forty-five-months follow-up of a minimally invasive, interdisciplinary treated hemangioma of the mandible with a high risk of severe bleeding - a case report. Head Face Med. 2023;19(1):1.	Excluded; case report / series
179	Otonari-Yamamoto M, Nakajima K, Sato H, Wada H, Matsumoto H, Nishiyama A, et al. Dentigerous cysts suspected the other odontogenic lesions on panoramic radiography and CT. Oral Radiology. 2024;40(2):319-26.	Excluded; case report / series
180	Ozcan E, Ural A, Oktemer TK, Alpaslan G. Bilateral tonsillolithiasis: a case report. Oral Surg Oral Med Oral Pathol Oral Radiol Endod. 2006;102(3):e17-8.	Excluded; case report / series
181	Pace C, Ward S. Incidental finding of sialolithiasis in the sublingual gland: a diagnostic dilemma. Dent Update. 2011;38(10):704-5.	Excluded; case report / series
182	Padmavathi S, Anuradha G, Kannan A, Krithika CL. A Curious Case Report of "Nosey" Calcification. JOURNAL OF INDIAN ACADEMY OF ORAL MEDICINE AND RADIOLOGY. 2024;36(4):478-81.	Excluded; case report / series
183	Parker K, Visram S, Hodges S. An incidental finding of a long-standing button battery in the floor of the nose during a routine orthodontic examination. J Orthod. 2016;43(2):147-50.	Excluded; case report / series
184	Patil K, Sanjay CJ, Solayappan E, Suresh N. Facial, lingual, and infraorbital artery calcification: A rare incidental radiographic finding. European Journal of Anatomy. 2023;27(3):361-6.	Excluded; case report / series
185	Patil SR, Yadav N, Shailaja S, Dahal S, Misra N, Alam MK. Mandibular ameloblastoma: Report of three cases and a brief literature review. International Medical Journal. 2019;26(1):48-50.	Excluded; case report / series
186	Patinen P, Hietanen J, Peltola J. Sphenoid sinus mucocele: case report of an appearance on a panoramic radiograph. Oral Surg Oral Med Oral Pathol Oral Radiol Endod. 2002;93(6):747-50.	Excluded; case report / series
187	Pausch NC, Halama D. [Chance finding of a radiopaque lesion in the region of the left maxillary sinus]. Hno. 2014;62(10):756-8.	Excluded; case report / series
188	Payne K, Dickenson A. Worth syndrome as a diagnosis for mandibular osteosclerosis. Dentomaxillofac Radiol. 2011;40(8):531-3.	Excluded; case report / series
189	Pierse J, Ying-Peng Wun E, Pellecchia R, Wollenberg J. Treatment of a rare ganglioneuroma with resection and reconstruction of the mandible: A case report and literature review. Journal of Oral and Maxillofacial Surgery. 2014;72(4):748-.e9.	Excluded; case report / series
190	Pino Sáez N, Schilling Lara J, Schilling Quezada A, Celis Contreras C, Quintanilla Sfeir M, Hidalgo Rivas A. Atypical incidental finding in panoramic radiography of an extensive calcified mass in torus tubarius: a 11-year follow-up. Avances en Odontoestomatologia. 2023;39(1):17-23.	Excluded; case report / series
191	Polo M. Bone resorption under chin implants: The orthodontist's role in its diagnosis and management. Am J Orthod Dentofacial Orthop. 2017;151(1):201-8.	Excluded; case report / series
192	Ponraj RR, Sarah Samson R, Nayak VS, Mathew M. Unilateral short root anomaly: an incidental finding on routine radiographic examination. BMJ Case Rep. 2021;14(1).	Excluded; case report / series
193	Pradillos Garcés A, Gonzalez Lagunas J, Martínez Fuster X, Mareque Bueno J, Raspall Martín G, Umbert Massegur H. Aspergilosis simulando cuerpo extraño intrasinusal. Rev esp cir oral maxilofac. 2006;28(2):127-30.	Excluded; case report / series
194	Qureshi B. Incidental findings. Br Dent J. 2006;201(11):689.	Excluded; case report / series
195	Rajathi M, Austin RD, Mathew P, Bharathi CS, Srivastava KC. Autosomal-dominant osteopetrosis: an incidental finding. Indian J Dent Res. 2010;21(4):611-4.	Excluded; case report / series
196	Rajesh E, Dhivyadharshini S, Babu NA, Ramya C. The Unforeseen Revelation of Dentigerous Cyst. Journal of Pharmacy and Bioallied Sciences. 2024;16(Suppl 5):S4921-S4.	Excluded; case report / series
197	Ramesh A, Pabla T. Incidental finding on dental radiographs: benign fibro-osseous lesions of the jaws. J Mass Dent Soc. 2008;57(3):60-1.	Excluded; case report / series
198	Ramesh A, Pabla T. Incidental finding on dental radiographs: odontoma. J Mass Dent Soc. 2009;57(4):40-1.	Excluded; case report / series
199	Ramesh R, Siroraj P, Dcruz TM, Jeyapriya MV. Intraosseous schwannoma of mandible: a rare entity as an incidental finding on an orthopantomogram. BMJ Case Rep. 2022;15(6).	Excluded; case report / series
200	Reis G, Feltrin de Souza J, Santos DEN, de Menezes J, Fraiz FC, Koubik A, et al. Unexpected formation of root-like structures subsequent to the avulsion of immature permanent teeth: Case report. Dent Traumatol. 2021;37(4):647-52.	Excluded; case report / series
201	Rodrigues PA, Subramanya AP, Prabhuji MLV, Vardhan KB. Incidental finding of paradental cyst in the maxillary anterior teeth during immediate implant placement. Journal of Indian Society of Periodontology. 2022;26(6):614-8.	Excluded; case report / series
202	Saini T, Keene JJ, Whetten J. Radiographic diagnosis of supernumerary premolars: Case reviews. Journal of Dentistry for Children. 2002;69(2):184-90.	Excluded; case report / series
203	Sanjay CJ, Patil K, Jaishankar HP, Christopher VS. Unusually Pronounced and Expansive Mandibular Foramen. A Incidental Radiological Finding. International Journal of Morphology. 2024;42(3):549-53.	Excluded; case report / series
204	Sathish HT, Kamat S, Kamat M, Puranik RS. Schizodontic Molarization of Mandibular Premolars: A Case Report of Gemination. Ethiop J Health Sci. 2024;34(1):101-4.	Excluded; case report / series
205	Schafer T, Riggs B, Murakaru J, Kalathingal S. Incidental Finding of a Foreign Object on a Panoramic Radiograph. Pediatr Dent. 2015;37(5):453-4.	Excluded; case report / series
206	Schiroli G, Valente P, Valente F, Sbrenna L, Sbrenna A. Dental Panoramic Radiographs as Opportunistic Screening for Carotid Calcifications: A Case-Based Review. Cureus. 2025;17(10):e94585-e.	Excluded; case report / series
207	Selvi F, Cakarer S, Keskin C, Ozyuvaci H. Delayed removal of a maxillary third molar accidentally displaced into the infratemporal fossa. J Craniofac Surg. 2011;22(4):1391-3.	Excluded; case report / series
208	Shah SJ, Tandon S, Ratnani C, Sonwani I, Bishal J. Calcifying Epithelial Odontogenic Cysts of the Anterior Maxilla: Report of Two Cases. Cureus. 2024;16(7):e65392-e.	Excluded; case report / series
209	Sharif MO, Horner K, Chadwick S, West C. Susuk charms? A case report. Br Dent J. 2013;215(1):13-5.	Excluded; case report / series
210	Sibanda L, Wates E, Higginson J. Bony cystic lesion with associated submandibular lymphadenopathy on a background of breast carcinoma: an unexpected case of cervicofacial actinomycosis. BMJ Case Rep. 2020;13(4).	Excluded; case report / series
211	Sindi AM. Bilateral Mandibular Dentigerous Cysts Presenting as an Incidental Finding: A Case Report. Am J Case Rep. 2019;20:1148-51.	Excluded; case report / series
212	Singh P, Patil PM, Sawhney H, Patil SP, Mishra M. Giant tonsillolith: A rare oropharyngeal entity. Oral and Maxillofacial Surgery Cases. 2019;5(4).	Excluded; case report / series
213	Singhal I, Dave A, Arora M, Saluja P. Glandular odontogenic cyst - An unusual presentation in the maxilla. Journal of Clinical and Diagnostic Research. 2021;15(10):ZD18-ZD21.	Excluded; case report / series
214	Sitaraman P, Nagi R, Muthukrishnan A. Melorheostosis: The Incidental Finding of a Rare Bone Dysplasia in the Mandible on Panoramic and CBCT Images - A Case Report. JOURNAL OF INDIAN ACADEMY OF ORAL MEDICINE AND RADIOLOGY. 2024;36(4):457-9.	Excluded; case report / series
215	Snelgrove RA. Generalized idiopathic apical root resorption as an incidental finding in an adolescent: a case history. Dent Update. 1995;22(7):276-8.	Excluded; case report / series
216	Sophie K, Vivian T, Sarina M, Vanessa F, Mirco S, Christoph A, et al. Endoscopic-endonasal extraction of an ectopic tooth located in the left nasal cavity-a case report. Laryngo- Rhino- Otologie. 2022;101:S332-S3.	Excluded; case report / series
217	Steier L, Steier G, Doğramaci EJ, Rossi-Fedele G. Maxillary sinus unilateral aplasia as an incidental finding following cone-beam computed (volumetric) tomography. Aust Endod J. 2014;40(1):26-31.	Excluded; case report / series
218	Stoopler ET, Pinto A, Stanton DC, Mupparapu M, Sollecito TP. Extensive pneumatization of the temporal bone and articular eminence: an incidental finding in a patient with facial pain. Case report and review of literature. Quintessence Int. 2003;34(3):211-4.	Excluded; case report / series
219	Sun L, Sun Z, Ma X. Intracranial arachnoid cyst on dental radiography: a diagnostic challenge. Oral Surg Oral Med Oral Pathol Oral Radiol. 2013;115(3):e53-9.	Excluded; case report / series
220	Sundaresan PD, Foo M. Incidental finding of monoclonal B-cell lymphocytosis in the mandible. BMJ Case Rep. 2020;13(9).	Excluded; case report / series
221	Syed AZ, Jadallah B, Uzcategui M, Shaik SM. CBCT Diagnosis of a Stafne Bone Defect. J Mich Dent Assoc. 2017;99(4):44-6, 75.	Excluded; case report / series
222	Szalma J, Janovics K, Pacheco A, Kaszas B, Lempel E. Pre-eruptive intracoronal resorption in “high-risk” impacted third molars: A report of four cases. Journal of Cranio-Maxillofacial Surgery. 2022;50(10):798-805.	Excluded; case report / series
223	Tar I, Végh E, Martos R, Soós B, Márton I, Szekanecz Z. Six-Month Follow-Up of Periodontal Condition in Rheumatoid Arthritis and Ankylosing Spondylitis Arthritis Patients Undergoing Anti-Tumour Necrosis Factor-α Therapy. Journal of Clinical Medicine. 2024;13(2).	Excluded; case report / series
224	Tay AB. Long-standing intranasal foreign body: an incidental finding on dental radiograph: a case report and literature review. Oral Surg Oral Med Oral Pathol Oral Radiol Endod. 2000;90(4):546-9.	Excluded; case report / series
225	Tejasvi A, Chatra L, Prashanth Shenai K. Bilateral bifid mandibular condyle. Journal of Clinical and Diagnostic Research. 2011;5(2):390-2.	Excluded; case report / series
226	Treil J, Caron P, Nabet C. Pituitary adenoma as an incidental finding in dental radiology: A case report. Annals of Internal Medicine. 2014;160(4):290-1.	Excluded; case report / series
227	Trijolet JP, Parmentier J, Sury F, Goga D, Mejean N, Laure B. Cemento-ossifying fibroma of the mandible. Eur Ann Otorhinolaryngol Head Neck Dis. 2011;128(1):30-3.	Excluded; case report / series
228	Vasanthi V, Tha TP, Kumar AR, Balasubramanian S. Paradental cyst with hyaline ring granuloma masquerading as pericoronitis. Journal of Indian Society of Periodontology. 2023;27(4):434-6.	Excluded; case report / series
229	Vettori E, Borella A, Costantinides F, Rizzo R, Maglione M. Mandibular metastasis of pulmonary adenocarcinoma: How unexpected could it be? Gerodontology. 2024;41(2):283-8.	Excluded; case report / series
230	Vinay C, Uloopi KS, Rao RC, Kumar RS, Madhuri V. Oligodontia associated with osteopetrosis: a rare case report. J Dent Child (Chic). 2011;78(1):53-6.	Excluded; case report / series
231	Wakae-Morita S, Igarashi C, Osano T, Shimoda S, Kobayashi K. A case of a foreign body in the external auditory canal incidentally detected on magnetic resonance imaging. Oral Radiology. 2013;29(2):183-7.	Excluded; case report / series

232	Warhekar AM, Wanjari PV, Phulambrikar T. Unilateral trifid mandibular condyle: a case report. Cranio. 2011;29(1):80-4.	Excluded; case report / series
233	Winterboer J, Sander B, Korn P, Jehn P, Spalthoff S. Paediatric gingival extra-axial chordoma: an extremely rare entity. International Journal of Oral and Maxillofacial Surgery. 2022;51(12):1525-9.	Excluded; case report / series
234	Wölner-Hanssen AB, Pozzi E, Vajtai I, Bornstein MM. [Interradicular course of the inferior alveolar nerve at the mandibular right third molar and monostotic fibrous dysplasia affecting the mandibular left third molar in the same patient. A case report]. Schweiz Monatsschr Zahnmed. 2009;119(5):489-502.	Excluded; case report / series
235	Wong L, Khan S. Occult caries or pre-eruptive intracoronal resorption? A chance finding on a radiograph. Pediatr Dent. 2014;36(5):429-32.	Excluded; case report / series
236	Yıldızer EK, Gungor K, Kahraman SA. Cyst-like lesion in mandibular coronoid process: an unusual location. Oral Surg Oral Med Oral Pathol Oral Radiol. 2017;123(4):414-8.	Excluded; case report / series
237	Zaccheo F, Petroni G, Fiorillo L, Heboyan A, Carnevale G, Calapaj M, et al. Exceptional bone remodeling over composite restoration in the management of external root resorption: A case report. SAGE Open Medical Case Reports. 2024;12.	Excluded; case report / series
238	Zanganah YZ. Incidental finding. Br Dent J. 2004;197(2):62.	Excluded; case report / series
239	Zangoei Booshehri M, Pakshir HR, Fasihinia H, Hamedani S. Isolated bilateral molariform mandibular second premolars; a case report. BMC Oral Health. 2025;25(1):10.	Excluded; case report / series
240	Çukurovayilmaz Z, Tekin A. Relationship between the prevalence of soft tissue radiopacities on panoramic radiographs and medical conditions. Minerva Stomatologica. 2020;69(4):235-44.	Excluded; missing fulltext
241	Douilly G, Fouroux MA, Torres JH. [Radio-opaque masses detected on orthopantomograph]. Rev Stomatol Chir Maxillofac Chir Orale. 2013;114(6):e17-8.	Excluded; missing fulltext
242	Webb G, Webb G, Parmar N, Dhariwal D. EP045 Cherubism: An incidental finding leading to an MDT investigative approach. 2024. p. e18.	Excluded; missing fulltext
243	Ashoori N, Ghorbanyjavadpour F, Rakhshan V. Associations between 44 nonmetric permanent dental traits or anomalies with skeletal sagittal malocclusions and sex, besides correlations across the variations or abnormalities. BMC ORAL HEALTH. 2022;22(1).	Excluded; no radiography
244	Akkitap MP, Gumru B. Can the Position of the Impacted Third Molars Be an Early Risk Indicator of Pathological Conditions? A Retrospective Cone-Beam Computed Tomography Study. JOMR-JOURNAL OF ORAL & MAXILLOFACIAL RESEARCH. 2023;14(2).	Excluded; other method
245	Aldosimani MA, Althumairy RI, Alzahrani A, Aljarbou FA, Alkatheeri MS, AlGhizzi MA, et al. The mid-mesial canal prevalence in mandibular molars of a Saudi population: A cone-beam computed tomography study. SAUDI DENTAL JOURNAL. 2020;33(7):581-6.	Excluded; other method
246	Becker A, Chaushu S, Casap-Caspi N. Cone-beam computed tomography and the orthosurgical management of impacted teeth. J Am Dent Assoc. 2010;141 Suppl 3:14s-8s.	Excluded; other method
247	Bruno IG, Bruno LV, Bachur MA. Hallazgo incidental en tomografía computada de haz cónico. Rev Ateneo Argent Odontol. 2020;62(1):25-30.	Excluded; other method
248	Bücher K, Galler M, Seitz M, Hickel R, Kunzelmann KH, Kühnisch J. Occlusal Caries Extension in Relation to Visual and Radiographic Diagnostic Criteria: Results from a Microcomputed Tomography Study. OPERATIVE DENTISTRY. 2015;40(3):255-62.	Excluded; other method
249	Chen CA, Ahn Y, Odell S, Mupparapu M, Graham DM. Developmental salivary gland depression in the ascending mandibular ramus: A cone-beam computed tomography study. Imaging Sci Dent. 2016;46(3):223-7.	Excluded; other method
250	da Silva E, Prado MC, Queiroz PM, Nejaim Y, Brasil DM, Groppo FC, et al. Assessing pulp stones by cone-beam computed tomography. CLINICAL ORAL INVESTIGATIONS. 2017;21(7):2327-33.	Excluded; other method
251	Haghanifar S, Ghobadi F, Vahdani N, Bijani A. Age estimation by pulp/tooth area ratio in anterior teeth using cone-beam computed tomography: comparison of four teeth. JOURNAL OF APPLIED ORAL SCIENCE. 2019;27.	Excluded; other method
252	Hermann L, Wenzel A, Schropp L, Matzen LH. Impact of CBCT on treatment decision related to surgical removal of impacted maxillary third molars: does CBCT change the surgical approach? DENTOMAXILLOFACIAL RADIOLOGY. 2019;48(8).	Excluded; other method
253	Kato C, Barra SG, Amaral TMP, Silva TA, Abreu LG, Brasileiro CB, et al. Cone-beam computed tomography analysis of cemento-osseous dysplasia-induced changes in adjacent structures in a Brazilian population. CLINICAL ORAL INVESTIGATIONS. 2020;24(8):2899-908.	Excluded; other method
254	Krzyzostaniak J, Surdacka A, Kulczyk T, Dyszkiewicz-Konwinska M, Owecka M. Diagnostic Accuracy of Cone Beam Computed Tomography Compared with Intraoral Radiography for the Detection of Noncavitated Occlusal Carious Lesions. CARIES RESEARCH. 2014;48(5):461-6.	Excluded; other method
255	Lambrianidis T, Lyroudia K, Pandelidou O, Nicolaou A. Evaluation of periapical radiographs in the recognition of C-shaped mandibular second molars. INTERNATIONAL ENDODONTIC JOURNAL. 2001;34(6):458-62.	Excluded; other method
256	Matzen LH, Villefrance JS, Norholt SE, Bak J, Wenzel A. Cone beam CT and treatment decision of mandibular third molars: removal vs. coronectomy-a 3-year audit. DENTOMAXILLOFACIAL RADIOLOGY. 2020;49(3).	Excluded; other method
257	Minowa K, Inoue N, Sawamura T, Matsuda A, Totsuka Y, Nakamura M. Evaluation of static bone cavities with CT and MRI. Dentomaxillofac Radiol. 2003;32(1):2-7.	Excluded; other method
258	Nedunchezhian K, Aswath N. Radio-diagnostic dilemmas of submandibular calcification-an incidental CBCT finding. Journal of Clinical and Diagnostic Research. 2018;12(9):ZD06-ZD8.	Excluded; other method
259	Ogawa A, Fukuta Y, Nakasato H, Nakasato S. Evaluation by dental cone-beam computed tomography of the incidence and sites of branches of the inferior dental canal that supply mandibular third molars. BRITISH JOURNAL OF ORAL & MAXILLOFACIAL SURGERY. 2016;54(10):1116-20.	Excluded; other method
260	Pette GA, Norkin FJ, Ganeles J, Hardigan P, Lask E, Zfaz S, et al. Incidental findings from a retrospective study of 318 cone beam computed tomography consultation reports. International Journal of Oral and Maxillofacial Implants. 2012;27(3):595-603.	Excluded; other method
261	Pires CA, Bissada NF, Becker JJ, Kanawati A, Landers MA. Mandibular Incisive Canal: Cone Beam Computed Tomography. CLINICAL IMPLANT DENTISTRY AND RELATED RESEARCH. 2012;14(1):67-73.	Excluded; other method
262	Rege ICC, Botelho TD, Martins AFL, Leles CR, Mendonça EF. Pixel gray measurement for the diagnosis of dental ankylosis in cone beam computed tomography images. ORAL SURGERY ORAL MEDICINE ORAL PATHOLOGY ORAL RADIOLOGY. 2021;131(6):721-9.	Excluded; other method
263	Sygouros A, Motro M, Ugurlu F, Acar A. Surgically assisted rapid maxillary expansion: Cone-beam computed tomography evaluation of different surgical techniques and their effects on the maxillary dentoskeletal complex. AMERICAN JOURNAL OF ORTHODONTICS AND DENTOFACIAL ORTHOPEDICS. 2014;146(6):748-57.	Excluded; other method
264	Vyas R, Gohel A. Trifurcated Mental Foramina: A Cone-Beam Computed Tomography Incidental Finding During the Implant Treatment Planning. Cureus. 2023;15(1):e33828-e.	Excluded; other method
265	Wakoh M, Goto TK, Matsuzaka K, Shibahara T, Kamio T. Sialo-cutaneous fistula with ectopic submandibular gland sialolith, revealing a hidden ipsilateral enlarged and elongated styloid process: a consideration based on CT findings. Oral Radiology. 2021;37(2):336-44.	Excluded; other method
266	Yesiltepe S, Kilci G. Evaluation the relationship between the position and impaction level of the impacted maxillary third molar teeth and marginal bone loss, caries and resorption findings of the second molar teeth with CBCT scans. ORAL RADIOLOGY. 2022;38(2):269-77.	Excluded; other method
267	Yildirim EA, Türker N, Bulut DG, Ustaoglu G. The relationship of the position of mandibular third molar impaction with the development of dental and periodontal lesions in adjacent second molars. JOURNAL OF STOMATOLOGY ORAL AND MAXILLOFACIAL SURGERY. 2024;125(1).	Excluded; other method
268	Aktan AM, Kara I, Sener I, Bereket C, Çelik S, Kirtay M, et al. An evaluation of factors associated with persistent primary teeth. EUROPEAN JOURNAL OF ORTHODONTICS. 2012;34(2):208-12.	Excluded; single finding
269	Al-Khateeb TH, Bataineh AB. Pathology associated with impacted mandibular third molars in a group of Jordanians. JOURNAL OF ORAL AND MAXILLOFACIAL SURGERY. 2006;64(11):1598-602.	Excluded; single finding
270	Almog DM, Horev T, Illig KA, Green RM, Carter LC. Correlating carotid artery stenosis detected by panoramic radiography with clinically relevant carotid artery stenosis determined by duplex ultrasound. Oral Surgery, Oral Medicine, Oral Pathology, Oral Radiology, and Endodontics. 2002;94(6):768-73.	Excluded; single finding
271	Azhar A, Yusof NAA, Sugarman MK, Reduwan NH. Idiopathic Osteosclerosis among UiTM Dental Centre Patients: A Retrospective Study. ARCHIVES OF OROFACIAL SCIENCE. 2024;19(1):47-56.	Excluded; single finding
272	Boffano P, Cavarra F, Brucoli M, Ruslin M, Forouzanfar T, Ridwan-Pramana A, et al. The epidemiology and management of odontomas: a European multicenter study. Oral Maxillofac Surg. 2023;27(3):479-87.	Excluded; single finding
273	Cavalcante IL, Bezerra HIdO, Gonzaga AKG, Moreira-Souza L, Cral WG, de-Oliveira PT, et al. Radiographic evaluation of the prevalence of Stafne bone defect. Int j odontostomatol (Print). 2020;14(3):348-53.	Excluded; single finding
274	Celik Ozsoy S, Zirek T, Bahrilli S, Yuksel IB, Altindag A. Examination of the Frequency of Soft Tissue Ossification and Calcifications in Panoramic Radiographs: A Retrospective Study. Diagnostics. 2025;15(16).	Excluded; single finding
275	Cocos DI, Bud E, Pacurar M, Galea C, Alina C, Earar K. CLINICAL-STATISTICAL STUDY ON THE FREQUENCY OF WISDOM TOOTH INCLUSION PATHOLOGY. ROMANIAN JOURNAL OF ORAL REHABILITATION. 2024;16(4):840-51.	Excluded; single finding
276	Constantine S, Roach D, Liberali S, Kiermeier A, Sarkar P, Jannes J, et al. Carotid Artery Calcification on Orthopantomograms (CACO Study) – is it indicative of carotid stenosis? Australian Dental Journal. 2019;64(1):4-10.	Excluded; single finding
277	Fardi A, Kondylidou-Sidira A, Bachour Z, Parisis N, Tsirlis A. Incidence of impacted and supernumerary teeth-a radiographic study in a North Greek population. MEDICINA ORAL PATOLOGIA ORAL Y CIRUGIA BUCAL. 2011;16(1):E56-E61.	Excluded; single finding
278	Friedlander AH, Cohen SN. Panoramic radiographic atheromas portend adverse vascular events. Oral Surg Oral Med Oral Pathol Oral Radiol Endod. 2007;103(6):830-5.	Excluded; single finding
279	Friedrich RE, Scheuer HA, Fuhrmann A, Zustin J, Assaf AT. Radiographic findings of odontogenic myxomas on conventional radiographs. Anticancer Res. 2012;32(5):2173-7.	Excluded; single finding
280	Garbelotti-Junior S, Ribeiro-Moreira W, Aguiar H, Olave E, Queiroz D. Prevalencia de lesiones ateroscleróticas carotídeas en radiografía panorámica y su relación con factores de riesgo cardiovascular en brasileños. Int j morphol. 2020;38(6):1560-5.	Excluded; single finding
281	Ghassemzadeh S, Sbricoli L, Frigo AC, Bacci C. Incidental findings detected with panoramic radiography: prevalence calculated on a sample of 2017 cases treated at a major Italian trauma and cancer centre. Oral Radiol. 2021;37(3):507-17.	Excluded; single finding
282	Ghazali AB, Arayasantiparb R, Juengsomjit R, Lam-Ubol A. Central Odontogenic Myxoma: A Radiographic Analysis. International Journal of Dentistry. 2021;2021.	Excluded; single finding
283	Gomes ACA, Vasconcelos B, Silva E, Caldas ADF, Neto ICP. Sensitivity and specificity of pantomography to predict inferior alveolar nerve damage during extraction of impacted lower third molars. JOURNAL OF ORAL AND MAXILLOFACIAL SURGERY. 2008;66(2):256-9.	Excluded; single finding
284	Guerra Cobián O, Fuentes Puebla LL, Felipe Torres S. Lesiones radiopacas en tejido blando bucofacial. Comportamiento clínico-epidemiológico y manejo terapéutico en pacientes implantológicos. Rev habanera cienc méd. 2016;15(5):0-.	Excluded; single finding
285	Gujjar SV, Aggarwal A, Upadhyay N, Rathore A, Bisht J, Astekar M. Radiographic Evaluation of Tonsilloliths in Digital Panoramic Radiography - An Institutional-Based Retrospective Study. JOURNAL OF INDIAN ACADEMY OF ORAL MEDICINE AND RADIOLOGY. 2023;35(4):572-6.	Excluded; single finding
286	Gulsahi A, Cebeci AI, Özden S. A radiographic assessment of the prevalence of pulp stones in a group of Turkish dental patients. INTERNATIONAL ENDODONTIC JOURNAL. 2009;42(8):735-9.	Excluded; single finding
287	Gupta D, Sheikh S, Pallagatti S, Aggarwal A, Goyal G, Chidanandappa RN, et al. Zygomatic air cell defect: a panoramic radiographic study of a North Indian population. Journal of Investigative and Clinical Dentistry. 2013;4(4):247-51.	Excluded; single finding
288	Hajmohammadi E, Najirad S, Mikaeili H, Kamran A. Epidemiology of Supernumerary Teeth in 5000 Radiography Films: Investigation of Patients Referring to the Clinics of Ardabil in 2015-2020. INTERNATIONAL JOURNAL OF DENTISTRY. 2021;2021.	Excluded; single finding

289	Hvaring CL, Ogaard B, Stenvik A, Birkeland K. The prognosis of retained primary molars without successors: infraocclusion, root resorption and restorations in 111 patients. EUROPEAN JOURNAL OF ORTHODONTICS. 2014;36(1):26-30.	Excluded; single finding
290	Iwamoto Y, Uchida K, Sugino N, Kuroiwa H, Kitamura Y, Udagawa N, et al. Osteoporosis, osteoporotic fractures, and carotid artery calcification detected on panoramic radiographs in Japanese men and women. Oral Surg Oral Med Oral Pathol Oral Radiol. 2016;121(6):673-80.	Excluded; single finding
291	Izzetti R, Gennai S, De Francesco P, Carli E, Graziani F, Nisi M. Panoramic Radiography Features of Medication-Related Osteonecrosis of the Jaws (MRONJ). International Journal of Dentistry. 2023;2023.	Excluded; single finding
292	Johansson EP, Ahlqvist J, Garoff M, Karp K, Jäghagen EL, Wester P. Ultrasound screening for asymptomatic carotid stenosis in subjects with calcifications in the area of the carotid arteries on panoramic radiographs: a cross-sectional study. BMC Cardiovasc Disord. 2011;11:44.	Excluded; single finding
293	Kashyap RR, Kashyap RS, Kini R, Naik V. Prevalence of hyperdontia in nonsyndromic South Indian population: An institutional analysis. Indian J Dent. 2015;6(3):135-8.	Excluded; single finding
294	Kumar V, Kumar S, Kumar M, Saba R. A retrospective assessment of the radiographic records to analyze the prevalence of supernumerary teeth among non-syndromic patients. European Journal of Molecular and Clinical Medicine. 2020;7(10):4362-7.	Excluded; single finding
295	Kuna T, Vuletić M, Gabrić D. GORLIN-GOLTZ SYNDROME - TREATMENT STAGES. Acta Stomatologica Croatica. 2024;54(4):396.	Excluded; single finding
296	Mahdian M, Moghaddam EJ, Alzahrani A, Rengasamy K, Tadinada A. Calcification of the stylohyoid ligament in panoramic radiography and cone beam computed tomography among patients referred for dental implant treatment planning. Implant Dent. 2014;23(4):508-13.	Excluded; single finding
297	Miloglu O, Yalcin E, Buyukkurt M, Yilmaz A, Harorli A. The frequency of bifid mandibular condyle in a Turkish patient population. Dentomaxillofac Radiol. 2010;39(1):42-6.	Excluded; single finding
298	Monteiro IA, Ibrahim C, Albuquerque R, Donaldson N, Salazar F, Monteiro L. Assessment of carotid calcifications on digital panoramic radiographs: Retrospective analysis and review of the literature. Journal of Stomatology, Oral and Maxillofacial Surgery. 2018;119(2):102-6.	Excluded; single finding
299	Nelke K, Matys J, Janeczek M, Malyszek A, Luczak K, Lukaszewski M, et al. The Occurrence and Outcomes of Cemento-Osseous Dysplasias (COD) in the Jaw Bones of the Population of Lower Silesia, Poland. J Clin Med. 2024;13(22).	Excluded; single finding
300	Niknami M, Mirmohammadi M, Pezeshki A. Evaluation of the Prevalence of Mucous Retention Pseudocyst and its Correlation with the Associated Risk Factors Using Panoramic Radiography and Cone-Beam Computed Tomography. J Dent (Tehran). 2018;15(2):123-9.	Excluded; single finding
301	Ohba T, Takata Y, Ansai T, Morimoto Y, Tanaka T, Kito S, et al. Evaluation of calcified carotid artery atheromas detected by panoramic radiograph among 80-year-olds. Oral Surgery, Oral Medicine, Oral Pathology, Oral Radiology, and Endodontics. 2003;96(5):647-50.	Excluded; single finding
302	Patil SR. Prevalence of carotid artery calcification in postmenopausal women and its correlation with atherogenic risk factors. J Nat Sci Biol Med. 2015;6(Suppl 1):S1-6.	Excluded; single finding
303	Peker I, Kaya E, Darendeliler-Yaman S. Clinical and radiographical evaluation of non-syndromic hypodontia and hyperdontia in permanent dentition. MEDICINA ORAL PATOLOGIA ORAL Y CIRUGIA BUCAL. 2009;14(8):E393-E7.	Excluded; single finding
304	Ragu R, Jaiswal M, Rathore M, Goyal A, Kumar A, Kapur A. Frequency and characteristics of gnathic dense bone islands in a paediatric population residing in Chandigarh, India: a cross-sectional radiographic study. Eur Arch Paediatr Dent. 2025;26(1):57-62.	Excluded; single finding
305	Ribeiro A, Keat R, Khalid S, Ariyaratnam S, Makwana M, do Pranto M, et al. Prevalence of calcifications in soft tissues visible on a dental pantomogram: A retrospective analysis. Journal of Stomatology, Oral and Maxillofacial Surgery. 2018;119(5):369-74.	Excluded; single finding
306	Sahman H, Sekerci AE, Ertas ET, Etoz M, Sisman Y. Prevalence of bifid mandibular condyle in a Turkish population. J Oral Sci. 2011;53(4):433-7.	Excluded; single finding
307	Santos KK, Lages FS, Maciel CAB, Glória JCR, Douglas-de-Oliveira DW. Prevalence of Mandibular Third Molars According to the Pell & Gregory and Winter Classifications. JOURNAL OF MAXILLOFACIAL & ORAL SURGERY. 2022;21(2):627-33.	Excluded; single finding
308	Schaefer C, Pingel S, Ghanem A, Weisser-Thomas J, Doerner J, Helfgen E, et al. Calcification of the carotid artery territory in dental panoramic radiography is predictive for excessive cardiovascular risk. Vasa - European Journal of Vascular Medicine. 2014;43:53.	Excluded; single finding
309	Senosiain-Oroquieta A, Pardo-López B, De Carlos-Villafranca F, Cobo-Plana J. Detección de placas de ateroma mediante radiografías dentales. RCOE, Rev Ilustre Cons Gen Col Odontól Estomatól Esp. 2006;11(3):297-303.	Excluded; single finding
310	Sergio-Lins D-A-V, João-Victor-Pereira M, Teresa-Cristina-Rangel P, Deborah-Queiroz F. More frequent detection of calcified carotid atherosclerotic plaques and mineralized laryngeal cartilages on digital than on film-based panoramic radiographs. Imaging Science in Dentistry. 2019:65-70.	Excluded; single finding
311	Shah AM, Shah D, Butoliya P, Chakrabarty H, Verma I, Chaurasia I, et al. INCIDENCE OF MUCOUS RETENTION CYST IN MAXILLARY SINUS; A RETROSPECTIVE RADIOGRAPHIC STUDY. Journal of Pharmaceutical Negative Results. 2022;13:4093-7.	Excluded; single finding
312	Taguchi A, Ohtsuka M, Tsuda M, Nakamoto T, Kodama I, Inagaki K, et al. Risk of vertebral osteoporosis in post-menopausal women with alterations of the mandible. Dentomaxillofacial Radiology. 2007;36(3):143-8.	Excluded; single finding
313	Tarim-Ertas ET, Şişman Y. Detection of incidental carotid artery calcifications during dental examinations: Panoramic radiography as an important aid in dentistry. Oral Surgery, Oral Medicine, Oral Pathology, Oral Radiology, and Endodontics. 2011;112(4):e11-e7.	Excluded; single finding
314	Tercanlı H, Kurtuldu E, Bilge NH, Yilmaz S, Bilge OM. Environmental temperature and air humidity and prevalence of maxillary sinus retention cysts: possible relationships (preliminary study). Oral Radiology. 2019;35(3):296-300.	Excluded; single finding
315	Tkaczuk A, Perry K, Caccamese J, Ord R, Pereira K. Cystic lesions of the jaws in children: A 15 year experience. Otolaryngology - Head and Neck Surgery (United States). 2013;149(2):P242-P3.	Excluded; single finding
316	Uzun I, Gunduz K, Canitezer G, Avsever H, Orhan K. A retrospective analysis of prevalence and characteristics of pre-eruptive intracoronal resorption in unerupted teeth of the permanent dentition: a multicentre study. INTERNATIONAL ENDODONTIC JOURNAL. 2015;48(11):1069-76.	Excluded; single finding
317	Vallo J, Suominen-Taipale L, Huumonen S, Soikkonen K, Norblad A. Prevalence of mucosal abnormalities of the maxillary sinus and their relationship to dental disease in panoramic radiography: results from the Health 2000 Health Examination Survey. Oral Surg Oral Med Oral Pathol Oral Radiol Endod. 2010;109(3):e80-7.	Excluded; single finding
318	Ferrús-Torres E, Gargallo-Albiol J, Berini-Aytés L, Gay-Escoda C. Diagnostic predictability of digital versus conventional panoramic radiographs in the presurgical evaluation of impacted mandibular third molars. INTERNATIONAL JOURNAL OF ORAL AND MAXILLOFACIAL SURGERY. 2009;38(11):1184-7.	Excluded; no incidental finding
319	Patil S, Sinha N. Pulp stone, haemodialysis, end-stage renal disease, carotid atherosclerosis. Journal of Clinical and Diagnostic Research. 2013;7(6):1228-31.	Excluded; no incidental finding
320	Wiechens B, Klenke D, Quast A, Santander P, Skorna I, Meyer-Marcotty P. Radiodiagnostics of standard orthodontic radiographs-dental and extradental incidental findings : Do we see everything we should see? J Orofac Orthop. 2024;85(Suppl 2):1-11.	Excluded; no incidental finding
321	Benediktsdottir IS, Hintze H, Petersen JK, Wenzel A. Accuracy of digital and film panoramic radiographs for assessment of position and morphology of mandibular third molars and prevalence of dental anomalies and pathologies. DENTOMAXILLOFACIAL RADIOLOGY. 2003;32(2):109-15.	Excluded; localized assessment
322	Berasmo A, Sánchez J, Villarroel M. PREVALENCE OF PATHOLOGIES IN RETAINED MANDIBULAR THIRD MOLARS WITH ASSOCIATED RADIOLUCENT IMAGE IN PATIENTS OF THE POSTGRADUATE COURSE IN ORAL SURGERY AT THE CENTRAL UNIVERSITY OF VENEZUELA (2010-2019). ACTA BIOCLINICA. 2020;10(20):113-33.	Excluded; localized assessment
323	Berasmo A, Sánchez J, Villarroel M. PREVALENCE OF PATHOLOGIES IN RETAINED MANDIBULAR THIRD MOLARS WITH ASSOCIATED RADIOLUCENT IMAGE IN PATIENTS OF THE POSTGRADUATE COURSE IN ORAL SURGERY AT THE CENTRAL UNIVERSITY OF VENEZUELA (2010-2019). ACTA BIOCLINICA. 2021;11(21):185-205.	Excluded; localized assessment
324	Blakey GH, Marciani RD, Haug RH, Phillips C, Offenbacher S, Pabla T, et al. Periodontal pathology associated with asymptomatic third molars. J Oral Maxillofac Surg. 2002;60(11):1227-33.	Excluded; localized assessment
325	Herranz-Aparicio J, Marques J, Almendros-Marqués N, Gay-Escoda C. Retrospective study of the bone morphology in the posterior mandibular region. Evaluation of the prevalence and the degree of lingual concavity and their possible complications. MEDICINA ORAL PATOLOGIA ORAL Y CIRUGIA BUCAL. 2016;21(6):E731-E6.	Excluded; localized assessment
326	Kaeppler G. Conventional cross-sectional tomographic evaluation of mandibular third molars. QUINTESSENCE INTERNATIONAL. 2000;31(1):49-56.	Excluded; localized assessment
327	Khouri C, Aoun G, Khouri C, Saade M, Salameh Z, Berberi A. Evaluation of Third Molar Impaction Distribution and Patterns in a Sample of Lebanese Population. JOURNAL OF MAXILLOFACIAL & ORAL SURGERY. 2022;21(2):599-607.	Excluded; localized assessment
328	Skitioui M, Jaoui D, Khalaf LH, Touré B. Mandibular Second Molars and Their Pathologies Related to the Position of the Mandibular Third Molar: A Radiographic Study. CLINICAL COSMETIC AND INVESTIGATIONAL DENTISTRY. 2023;15:215-23.	Excluded; localized assessment
329	Yildirim H, Büyükgöze-Dindar M. Investigation of the prevalence of impacted third molars and the effects of eruption level and angulation on caries development by panoramic radiographs. MEDICINA ORAL PATOLOGIA ORAL Y CIRUGIA BUCAL. 2022;27(2):E106-E12.	Excluded; localized assessment
330	Abdel-Kadir HM. Incidental findings of pathology and abnormality in pretreatment orthodontic panoramic radiographs. Angle Orthod. 2006;76(3):following 541; author reply.	Excluded; no clinical study
331	Aghdasi MM, Valizadeh S, Amin-Tavakoli N, Bakhshandeh H. Tonsilolith in Routine Panoramic Radiographies; Is It a Common incidental Finding? Iranian Journal of Radiology. 2012;9(2):71-2.	Excluded; no clinical study
332	Alves IdS, Vendramini DFV, Departamento de RadiologiaLeite CdC, Gebrim EMMS, Passos UL. Dental findings on face and neck imaging. Radiol bras. 2021;54(2):107-14.	Excluded; no clinical study
333	Belloni P, Pelliccioni M, Toniolo D, Candido P, Cozzi C, Bollina R. Denosumab (DMAB) and osteonecrosis of the jaw (ONJ). Tumori. 2020;106(2 SUPPL):202-3.	Excluded; no clinical study
334	Friedland B, Metson R. A Guide to Recognizing Maxillary Sinus Pathology and for Deciding on Further Preoperative Assessment Prior to Maxillary Sinus Augmentation. INTERNATIONAL JOURNAL OF PERIODONTICS & RESTORATIVE DENTISTRY. 2014;34(6):807-15.	Excluded; no clinical study
335	Friedler AH, Bertolami CN. The ethics of managing incidental findings: Implications and challenges for the profession. Journal of the American Dental Association. 2014;145(9):910-1.	Excluded; no clinical study
336	Fun CL, Jinn FY. BIFID MANDIBULAR CONDYLE. ORAL SURGERY ORAL MEDICINE ORAL PATHOLOGY ORAL RADIOLOGY AND ENDODONTOLOGY. 1990;69(1):24-7.	Excluded; no clinical study
337	Gawron K, Łazarz-Bartyzel K, Łazarz M, Potempa J, Chomyszyn-Gajewska M. Fibrotic fibrils content and the treatment outcome: A study of two siblings with hereditary gingival fibromatosis. Dental and Medical Problems. 2016;53(1):158-9.	Excluded; no clinical study
338	Isaacson KG, Thom AR. Incidental findings [1]. British Dental Journal. 2007;202(3):113.	Excluded; no clinical study
339	Ito K, Hirahara N, Muraoka H, Okada S, Kondo T, Andreu-Arasa VC, et al. Normal Variants of the Oral and Maxillofacial Region: Mimics and Pitfalls. Radiographics. 2022;42(2):506-21.	Excluded; no clinical study
340	Janardhanan M, Rakesh S, Savithri V, Aravind T, Mohan M. Carcinoma Cuniculatum of Mandible Masquerading as Odontogenic Keratocyst: Challenges in the Histopathological Diagnosis. Head Neck Pathol. 2021;15(4):1313-21.	Excluded; no clinical study
341	Kwiatkowski MI, Carter E, Makdissi J. The importance of correct patient positioning. Dental Update. 2015;42(3):294-5.	Excluded; no clinical study
342	Lyons AJ, Babajews AV. Gigantiform cementoma--an unusual incidental finding. Br J Radiol. 1986;59(699):277-9.	Excluded; no clinical study
343	Macanovic M, Gangidi S, Porter G, Brown S, Courtney D, Porter J. Incidental bony pathology when reporting trauma orthopantomograms. Clin Radiol. 2010;65(10):842-9.	Excluded; no clinical study
344	Math SY, Murugeshappa D, Turner TWS, Flores-Mir C, Pachêco-Pereira C. Orthodontic craniofacial imaging and systemic conditions: Radiographic indicators and clinical implications. Seminars in Orthodontics. 2025;31(4):456-63.	Excluded; no clinical study
345	Ng CH, Pr C, K P, Lfa S. The Clinical Significance Of The Incisive Foramen. 2025. p. 386.	Excluded; no clinical study

346	Rioux-Forker D, Deziel AC, Williams LS, Muzaffar AR. Odontogenic Cysts and Tumors. Ann Plast Surg. 2019;82(4):469-77.	Excluded; no clinical study
347	Sadda RS, Phelan J. Dental management of florid cemento-osseous dysplosia. N Y State Dent J. 2014;80(3):24-6.	Excluded; no clinical study
348	Sbirkova T, Cholakova R. Intraosseous hemangiomas of the jaw. General Medicine. 2015;16(3):54-6.	Excluded; no clinical study
349	Taguchi A, Tanaka R, Kakimoto N, Morimoto Y, Arai Y, Hayashi T, et al. Clinical guidelines for the application of panoramic radiographs in screening for osteoporosis. Oral Radiology. 2021;37(2):189-208.	Excluded; no clinical study
350	Terzic A, Becker M, Imholz B, Scolozzi P. Unilateral widening of the inferior alveolar nerve canal: A rare anatomic variant mimicking disease. Oral Radiology. 2013;29(2):160-5.	Excluded; no clinical study
351	Akalln A, Koç N, Özşin-Özler C, Karaosmanoglu B, Ürel-Demir G, Taskiran EZ, et al. A Novel Pathogenic TSPEAR Variant in a Family with Clinical Variability: Definition of Dental Anomalies and Review of the Literature. Molecular Syndromology. 2025;16(4):291-310.	Excluded; review
352	Almog DM, Illig KA, Carter LC, Friedlander AH, Brooks SL, Grimes RM. Diagnosis of non-dental conditions. Carotid artery calcifications on panoramic radiographs identify patients at risk for stroke. N Y State Dent J. 2004;70(8):20-5.	Excluded; review
353	Bohay RN, Gordon SC. The maxillary mucous retention cyst: a common incidental panoramic finding. Oral Health. 1997;87(7):7-8, 10.	Excluded; review
354	Aikins EA, Ututu C, Chukwuma EI. Prevalence of incidental dental anomalies seen on pre-treatment digital panoramic radiographs of a group of Nigerian orthodontic patients: A retrospective study. Niger J Dent Res (Online). 2022;7(1):67-74.	Included
355	Al-Abdallah M, AlHadidi A, Hammad M, Al-Ahmad H, Saleh R. Prevalence and distribution of dental anomalies: A comparison between maxillary and mandibular tooth agenesis. AMERICAN JOURNAL OF ORTHODONTICS AND DENTOFACIAL ORTHOPEDICS. 2015;148(5):793-8.	Included
356	Almeida FT, Gianoni-Capenakas S, Rabie H, Figueiredo R, Pacheco-Pereira C. The use of panoramic radiographs to address the oral health needs of vulnerable Canadian populations. Can J Dent Hyg. 2024;58(1):19-25.	Included
357	Bondemark L, Jeppsson M, Lindh-Ingildsen L, Rangne K. Incidental findings of pathology and abnormality in pretreatment orthodontic panoramic radiographs. Angle Orthod. 2006;76(1):98-102.	Included
358	Büyükgöze-Dindar M, Tekbas-Atay M. nZ Prevalence of Dental Anomalies Assessed Using Panoramic Radiographs in a Sample of the Turkish Population. CHINESE JOURNAL OF DENTAL RESEARCH. 2022;25(3):189-96.	Included
359	Cral WG. Achados incidentais em radiografias panorâmicas de pacientes pré e pós-tratamento ortodôntico. 2016. p. 95-.	Included
360	da Silva Dias ACM, De Medeiros AMC, de Freitas YNL, Costa-Lima KC, Maia PRL, Oliveira PT. Radiographic findings in panoramic radiographs of the elderly: Cross-sectional study in 1006 patients. Revista Portuguesa de Estomatologia, Medicina Dentaria e Cirurgia Maxilofacial. 2019;60(2):59-65.	Included
361	Edmonds M, Perschbacher S, Cioffi I, Magalhaes M. Ability of Orthodontists to Detect, Interpret and Propose Management Strategies for Incidental Findings on Pre-Treatment Panoramic Radiographs. Orthod Craniofac Res. 2025;28(3):449-57.	Included
362	Escolano Rivas J, Barrientos Sánchez S, Rodríguez Ciodaro A. Frecuencia y características de hallazgos y variaciones óseas en radiografías panorámicas de personas con edentulismo total. Univ odontol. 2018;37(78):1-10.	Included
363	Hernández G, Plaza SP, Cifuentes D, Villalobos LM, Ruiz LM. Incidental findings in pre-orthodontic treatment radiographs. Int Dent J. 2018;68(5):320-6.	Included
364	Hlongwa P, Moshaoa MAL, Musemwa C, Khammissa RAG. Incidental Pathologic Findings from Orthodontic Pretreatment Panoramic Radiographs. Int J Environ Res Public Health. 2023;20(4).	Included
365	Imanimoghaddam M, Tohidi E, Yazdi AA, Nikbakhsh E, Goudarzi F. Incidental findings in digital panoramic radiography of patients referred to mashhad dental school. Journal of Kerman University of Medical Sciences. 2021;28(1):43-55.	Included
366	Indra G, Maragathavalli G, Deepika R. Analysis of panoramic images of orthodontic patients. International Journal of Research in Pharmaceutical Sciences. 2020;11(Special Issue 3):1750-4.	Included
367	Jadu FM, Jan A. Incidental findings on panoramic radiographs for pre-extraction assessment of third molars. Asian J. Sci. Technol. 2015;6:1539-43.	Included
368	Jiménez Ortíz JL, Herrera Silva JC, Jiménez Ortíz JD, Pérez Lizárraga E, Murillo Ruiz JA. Hallazgos incidentales en ortopantomografías maxilomandibulares de pacientes adultos jóvenes. Rev ADM. 2017;74(1):25-31.	Included
369	Karkaç A, Paksoy T. Determining the Incidental Pathologic Findings (IPFs) on panoramic radiographs before orthodontic treatments: A retrospective study. Balkan Journal of Dental Medicine. 2024;28(2):91-8.	Included
370	Leyva Altamirano JL, Vargas Purecko MdlL. Hallazgos incidentales en radiografías panorámicas previas al tratamiento de ortodoncia. Acta odontol venez. 2011;49(3).	Included
371	MacDonald D, Yu W. Incidental findings in a consecutive series of digital panoramic radiographs. IMAGING SCIENCE IN DENTISTRY. 2020;50(1):53-64.	Included
372	Mohan R, Puranik CP, Kaci P, Moore T, Katechia B, Schulman GS, et al. Prescription of panoramic radiographs in children using age-based prevalence of dental anomalies and pathologies. International Journal of Paediatric Dentistry. 2024;34(2):125-34.	Included
373	Pakbaznejad Esmaeili E, Ekholm M, Haukka J, Waltimo-Sirén J. Type and location of findings in dental panoramic tomographs in 7-12-year-old orthodontic patients. Acta Odontol Scand. 2016;74(4):272-8.	Included
374	Pamadya S, Kurniati N. incidental findings in panoramic radiographs: a descriptive study of panoramic radiographs taken at X Hospital in Jambi City, Indonesia. Journal of Health and Dental Sciences 2025;4(3):277-288.	Included
375	Sadik E, Gökmenoglu C, Kara C. Complete Edentulism of Dental Patients in Northeastern Turkey: Prevalence and Radiographic Findings on Panoramic Radiographs. JOURNAL OF DENTISTRY INDONESIA. 2020;27(3):139-43.	Included
376	Temur KT. Incidental dental anomalies and jawbone pathologies in Turkish pediatric patients of the Eastern Mediterranean region. Annals of Clinical and Analytical Medicine. 2021;12(12):1320-4.	Included
377	Vaiciulis LAG, Cavalcanti SCXB, Chilvarquer I, Luz JGC. Occurrence of nonarticular incidental findings on panoramic radiographs in painful temporomandibular disorders. Int j odontostomatol (Print). 2020;14(2):213-9.	Included
378	Yoga S, Vishwanath R, Sitra G, Vandana, Baliah J, Kayalvizhi. Occult pathologies disclosed in panoramic radiographs-a retrospective study. Int J Curr Res 2017;9(4):49330-49333.	Included

Appendix 6. Assessment of methodological soundness / internal validity / reporting completeness of included studies.

Question	Aikins 2022	Al-Abdallah 2015	Almeida 2024	Bondemark 2006	Büyükgöze-Dindar 2022	Cral 2016	da Silva Dias 2019	Edmonds 2025	Escolano Rivas 2018
Are details given about the origin of the analyzed sample?	Yes	Yes	Yes	Yes	Yes	Yes	Yes	Yes	Yes
Is the acquisition timeframe of included panoramic radiographs given?	Yes	Yes	Yes	Yes	Yes	Yes	Yes	Yes	No
According to eligibility criteria, are included patients representative of the general healthy population, the target population of interest?	No	No	No	No	Yes	No	Yes	No	No
Was an acceptable sampling method (e.g., consecutive or random selection) used to recruit cases?"	No	No	No	Yes	No	No	No	No	No
Is the age of included patients reported?	Yes	Yes	Yes	Partly	No	Partly	Yes	Yes	Yes
Is the sex of included patients reported?	Yes	Yes	Yes	Yes	No	Yes	Yes	Yes	Yes
Is the ethnicity of included patients reported?	No	No	No	No	No	No	No	No	No
Is the reason for acquisition of panoramic radiographs reported?	Yes	Partly	Yes	Partly	No	Yes	No	Yes	No
Are inclusion / exclusion criteria of eligible patients reported?	Yes	Yes	Yes	Yes	No	Yes	Partly	Yes	No
According to the reason for panoramic radiograph acquisition, are included patients representative of the general population?	Partly	Partly	No	Partly	Partly	Partly	Partly	Partly	No
Do the authors state which area was included in the panoramic radiograph?	No	No	No	No	No	No	No	No	Yes
Are technical details about the acquired panoramic radiograph given?	No	No	No	Yes	No	No	Yes	No	No
Are panoramic radiographs evaluated by an experienced certified radiologist?	No	No	Yes	Yes	Yes	No	Yes	Yes	Yes
Are assessors unaware of the reason for panoramic radiograph acquisition?	No	No	Yes	No	No	No	No	No	No
Have efforts been put into place to make sure optimal assessing conditions exist and errors are minimized?	No	No	Yes	Yes	Yes	No	No	No	No
Were all panoramic radiographs assessed under the same conditions?	No	No	Yes	Yes	Yes	Partly	No	No	No
Have panoramic radiographs been assessed from at least two assessors independently?	Yes	Yes	No	Yes	Yes	No	No	No	No
Have findings been reported on both patient level and panoramic radiograph image level, if applicable (multiple findings per image clarification)?	No	No	Partly	No	Yes	No	No	Partly	No
In case categorizations / poolings of findings have been made, have also separate individual findings been reported?	Yes	Yes	Yes	Yes	Yes	Yes	Yes	Yes	Yes
Have all possible findings that could be found in the particular panoramic radiograph image been reported?	No	No	No	Yes	Yes	No	Yes	Yes	No
Has an appropriately large sample been analyzed?	No	Yes	Yes	Yes	Yes	Yes	No	No	No
Has the prevalence of observed findings been assessed?	Yes	Yes	Yes	Yes	Yes	Yes	Partly	No	No

Appendix 6. Assessment of methodological soundness / internal validity / reporting completeness of included studies (continued).

Question	Hernández 2018	Hlongwa 2023	Imanimoghaddam 2021	Indra 2020	Jadu 2015	Jiménez Ortíz 2017	Karkac 2024	Leyva Altamirano 2011	MacDonald 2020
Are details given about the origin of the analyzed sample?	Yes	Yes	Yes	Yes	Yes	Yes	Yes	Yes	Yes
Is the acquisition timeframe of included panoramic radiographs given?	Yes	Yes	Yes	Yes	Yes	No	Yes	Yes	No
According to eligibility criteria, are included patients representative of the general healthy population, the target population of interest?	No	No	Yes	No	No	No	No	No	Yes
Was an acceptable sampling method (e.g., consecutive or random selection) used to recruit cases?"	Yes	Yes	No	Yes	No	No	Yes	No	No
Is the age of included patients reported?	Yes	Yes	Yes	No	Yes	Yes	Partly	Partly	Partly
Is the sex of included patients reported?	Yes	Yes	Yes	Yes	Yes	Yes	Yes	Yes	Yes
Is the ethnicity of included patients reported?	No	No	No	No	No	No	No	No	No
Is the reason for acquisition of panoramic radiographs reported?	No	No	Yes	No	Yes	No	Yes	Yes	Yes
Are inclusion / exclusion criteria of eligible patients reported?	Yes	Yes	No	Yes	Yes	No	Yes	Yes	Yes
According to the reason for panoramic radiograph acquisition, are included patients representative of the general population?	Partly	Partly	No	Partly	No	No	Partly	Partly	Yes
Do the authors state which area was included in the panoramic radiograph?	No	No	No	No	No	No	No	No	No
Are technical details about the acquired panoramic radiograph given?	Yes	Yes	No	No	No	No	No	No	No
Are panoramic radiographs evaluated by an experienced certified radiologist?	No	No	Yes	No	Yes	No	No	Yes	No
Are assessors unaware of the reason for panoramic radiograph acquisition?	No	No	No	No	No	Yes	No	No	No
Have efforts been put into place to make sure optimal assessing conditions exist and errors are minimized?	Yes	Yes	No	No	No	Yes	No	Yes	Yes
Were all panoramic radiographs assessed under the same conditions?	Yes	Yes	No	No	No	Yes	No	Yes	Yes
Have panoramic radiographs been assessed from at least two assessors independently?	No	No	Yes	Yes	No	Yes	No	No	No
Have findings been reported on both patient level and panoramic radiograph image level, if applicable (multiple findings per image clarification)?	No	Partly	No	No	No	No	No	No	No
In case categorizations / poolings of findings have been made, have also separate individual findings been reported?	Yes	Yes	Yes	Yes	Yes	Yes	Yes	Yes	Yes
Have all possible findings that could be found in the particular panoramic radiograph image been reported?	Yes	No	Yes	No	No	Yes	Yes	No	Yes
Has an appropriately large sample been analyzed?	Yes	No	No	Yes	Yes	Yes	Yes	Yes	No
Has the prevalence of observed findings been assessed?	Yes	Yes	No	Yes	Yes	Yes	Yes	Yes	Partly

Appendix 6. Assessment of methodological soundness / internal validity / reporting completeness of included studies (continued).

Question	Mohan 2024	Pakbaznejad Esmaeili 2016	Pamadya 2025	Sadik 2020	Temur 2021	Vaiciulis 2020	Yoga 2017
Are details given about the origin of the analyzed sample?	Yes	Yes	Yes	Yes	Yes	Yes	Yes
Is the acquisition timeframe of included panoramic radiographs given?	Yes	Yes	Yes	Yes	No	No	Yes
According to eligibility criteria, are included patients representative of the general healthy population, the target population of interest?	Yes	No	Yes	No	No	No	Yes
Was an acceptable sampling method (e.g., consecutive or random selection) used to recruit cases?"	No	Yes	Yes	Yes	No	No	No
Is the age of included patients reported?	Partly	Partly	No	Yes	Partly	Yes	No
Is the sex of included patients reported?	Yes	Yes	No	Yes	Yes	Yes	No
Is the ethnicity of included patients reported?	No	No	No	No	No	No	No
Is the reason for acquisition of panoramic radiographs reported?	No	Partly	No	No	No	Yes	No
Are inclusion / exclusion criteria of eligible patients reported?	Yes	Yes	Yes	No	Yes	Yes	Partly
According to the reason for panoramic radiograph acquisition, are included patients representative of the general population?	Partly	Partly	Partly	No	Partly	No	No
Do the authors state which area was included in the panoramic radiograph?	No	Partly	Yes	No	No	No	No
Are technical details about the acquired panoramic radiograph given?	Yes	No	No	No	Yes	Yes	Partly
Are panoramic radiographs evaluated by an experienced certified radiologist?	Yes	Yes	No	Yes	Yes	No	No
Are assessors unaware of the reason for panoramic radiograph acquisition?	Yes	No	No	No	No	No	No
Have efforts been put into place to make sure optimal assessing conditions exist and errors are minimized?	Yes	No	Partly	No	No	No	No
Were all panoramic radiographs assessed under the same conditions?	Yes	No	Partly	No	No	No	No
Have panoramic radiographs been assessed from at least two assessors independently?	No	Yes	Yes	Yes	No	No	Yes
Have findings been reported on both patient level and panoramic radiograph image level, if applicable (multiple findings per image clarification)?	Partly	No	No	No	No	No	No
In case categorizations / poolings of findings have been made, have also separate individual findings been reported?	Yes	No	No	Yes	Yes	Yes	Yes
Have all possible findings that could be found in the particular panoramic radiograph image been reported?	Yes	No	Yes	No	No	Yes	Yes
Has an appropriately large sample been analyzed?	Yes	Yes	No	Yes	Yes	Yes	Yes
Has the prevalence of observed findings been assessed?	Yes	Yes	No	Yes	Yes	Yes	Yes

Appendix 7. Incidental findings from panoramic images reported from only one study and not included in the meta-analyses.

Nr	Finding	Prevalence
1	Endodontic treatment	8/250 (3.2%)
2	Mesiodens	52/1432 (3.6%)
3	Root tip embedded in bone	85/6252 (1.4%)
4	Sum: periodontal pathology	39/1032 (3.8%)
5	Third molar in mandibular canal	208/6252 (3.3%)

Appendix 8. Contour-enhanced funnel plot for the pooled prevalence for at least one incidental finding (Thompson P=0.14).

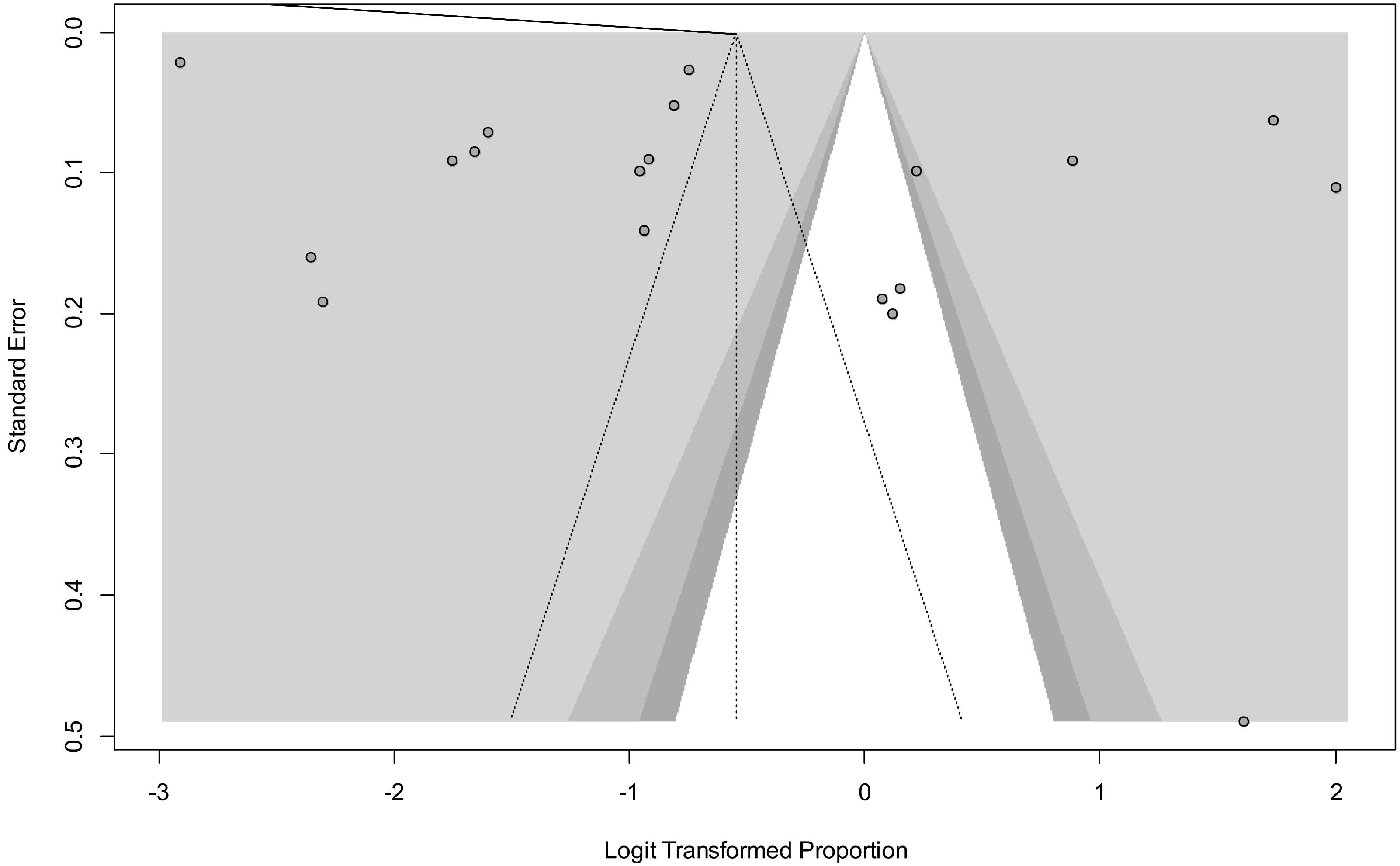

Appendix 9. Sensitivity analysis of pooled rate of incidental findings according to the sample size of included studies.

Nr	Number of patients	Pooled % rate (95% CI)	$p_{subsets}$
1	0-200	58.40 (39.33, 75.26)	0.006
2	201-500	20.70 (4.51, 59.08)	
3	501-1000	46.28 (13.41, 82.73)	
4	1001-2000	35.80 (5.82, 83.40)	
5	>2000	13.82 (0, 99.96)	

CI, confidence interval.
